# Fighting Antibiotic Resistance: New Pyrimidine-Clubbed Benzimidazole Derivatives as Potential DHFR Inhibitors

**DOI:** 10.3390/molecules28020501

**Published:** 2023-01-04

**Authors:** M. Akiful Haque, Akash Marathakam, Ritesh Rana, Samar J Almehmadi, Vishal B. Tambe, Manoj S. Charde, Fahadul Islam, Falak A. Siddiqui, Giulia Culletta, Anna Maria Almerico, Marco Tutone, Sharuk L. Khan

**Affiliations:** 1 Department of Pharmaceutical Analysis, School of Pharmacy, Anurag University, Ghatkesar, Hyderabad 500088, India; 2 National College of Pharmacy, Kozhikode 673602, India; 3 Department of Pharmaceutics, Himachal Institute of Pharmaceutical Education and Research (HIPER), Bela, Nadaun, Hamirpur 177042, India; 4 Department of Chemistry, Faculty of Applied Science, Umm-Al-Qura University, Makkah 24382, Saudi Arabia; 5 Department of Pharmaceutical Chemistry, Pravara Rural College of Pharmacy, Loni 413736, India; 6 Department of Pharmaceutical Chemistry, Government College of Pharmacy, Karad 415124, India; 7 Department of Pharmacy, Faculty of Allied Health Sciences, Daffodil International University, Dhaka 1207, Bangladesh; 8 Department of Pharmaceutical Chemistry, N.B.S. Institute of Pharmacy, Ausa 413520, India; 9 Dipartimento di Scienze e Tecnologie Biologiche Chimiche e Farmaceutiche, Università degli Studi di Palermo, 90123 Palermo, Italy

**Keywords:** DHFR, antifungal, antibacterial, pyrimidines, benzimidazoles, ADMETlab 2.0, molecular docking

## Abstract

The present work describes the design and development of seventeen pyrimidine-clubbed benzimidazole derivatives as potential dihydrofolate reductase (DHFR) inhibitors. These compounds were filtered by using ADMET, drug-likeness characteristics calculations, and molecular docking experiments. Compounds **27**, **29**, **30**, **33**, **37**, **38**, and **41** were chosen for the synthesis based on the results of the in silico screening. Each of the synthesized compounds was tested for its in vitro antibacterial and antifungal activities using a variety of strains. All the compounds showed antibacterial properties against Gram-positive bacteria (*Staphylococcus aureus* and *Staphylococcus pyogenes*) as well as Gram-negative bacteria (*Escherichia coli* and *Pseudomonas aeruginosa*). Most of the compounds either had a higher potency than chloramphenicol or an equivalent potency to ciprofloxacin. Compounds **29** and **33** were effective against all the bacterial and fungal strains. Finally, the 1,2,3,4-tetrahydropyrimidine-2-thiol derivatives with a 6-chloro-2-(chloromethyl)-1*H*-benzo[*d*]imidazole moiety are potent enough to be considered a promising lead for the discovery of an effective antibacterial agent.

## 1. Introduction

The treatment of nosocomial infections poses a significant global risk to public health due to drug-resistant bacteria, such as methicillin-resistant *Staphylococcus aureus* (MRSA) and multidrug-resistant *Escherichia coli* [1,2,3]. Research commissioned by the United Kingdom Government estimates that “the cost in terms of lost global production between now and 2050 would be an astounding 100 trillion USD” if no action is taken. Infections caused by fungi may significantly threaten human health, especially for immunocompromised patients. When it comes to clinical care, invasive fungal infections (IFIs) pose a significant challenge on a global scale [4,5,6]. It is imperative that attempts to discover new antibiotic agents be stepped up to keep pace with the worrisome increase in cases of antibiotic resistance being demonstrated by disease-causing microbes [7,8]. One crucial step is the identification of potent inhibitors of receptors that are critical to the bacteria’s survival.

One of these targets, known as dihydrofolate reductase (DHFR), may be found in the Gram-negative bacterium *Escherichia coli* and several microorganisms [9]. This enzyme is essential to the continued existence of the microbe. Since the middle of the 20th century, it has been shown that the DHFR enzyme may be used as a therapeutic target for the treatment of infections. DHFR plays a role in the production of raw materials for cell proliferation in both prokaryotic and eukaryotic cells. This is accomplished by catalyzing the reduction of dihydrofolate to tetrahydrofolate via the use of NADPH in the process [10]. Inhibitors of DHFR are employed extensively in the treatment of fungal infections, bacterial diseases, and mycobacterial diseases, as well as in the fight against malaria [11,12,13]. When it comes to finding novel and effective inhibitors for this enzyme, the traditional drug-discovery methods only depend on the rational drug design-based elaboration of core scaffolds.

The vast majority of DHFR inhibitors, which are either already in use or are the subject of research, are derivatives of folic acid that have a 2,4-diamino substitution in the pyrimidine ring. Structurally, these compounds belong to a variety of distinct classes (pyrimidines, pteridines, quinazolines, and pyrido-pyrimidines) [14,15]. It has been shown that several derivatives based on amino pyrimidine can inhibit DHFR, which in turn results in antibacterial activity [16,17]. Because of this, the pyrimidine scaffold was chosen for the current work to design and develop new DHFR inhibitors as possible antibacterial and antifungal medications. As an innovative approach, we have tried to merge the benzimidazole and pyrimidine nuclei in the hope of obtaining novel derivatives with synergistic effects. We aimed to obtain derivatives with a lower toxicity profile and improved biological potential. First, these new potential inhibitors were filtered by ADMET and drug-likeness calculations. Successively, molecular docking experiments were carried out. The compounds that showed substantial promise for DHFR inhibition were placed through a wet lab synthesis, which was then followed by a biological assessment.

## 2. Results and Discussion

### 2.1. In Silico ADMET Profile of Designed Molecules

**Figure 1:**
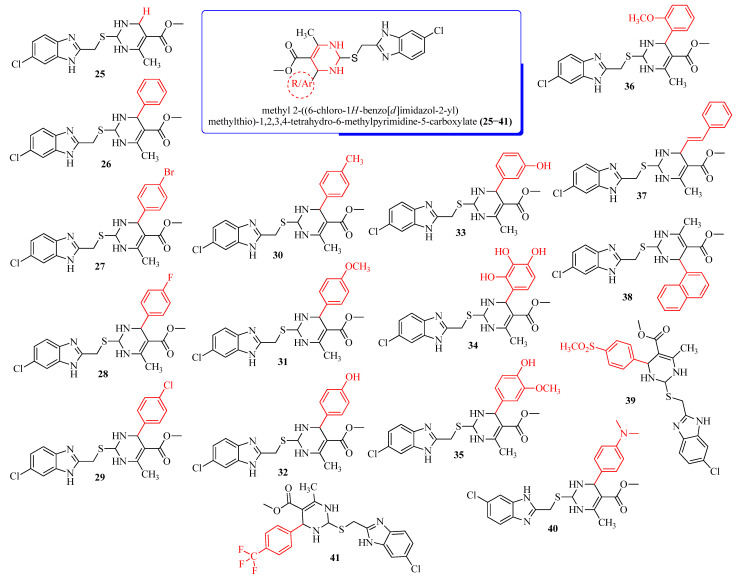
Parent nucleus and substitutions of methyl 2-((6-chloro-1*H*-benzo[*d*]imidazol-2-yl)methylthio)-1,2,3,4-tetrahydro-6-methylpyrimidine-5-carboxylate derivatives (**25**–**41**).

The ability for researchers to explore the biological effects of potential pharmacological candidates is made feasible by pharmacokinetic features, making them an essential part of the drug development process [18]. We designed a set of new pyrimidine-clubbed benzimidazole derivatives and performed ADMET, drug-like, and molecular docking calculations before starting the synthesis (Figure 1, 2D structures of the compounds). The physicochemical properties of the designed molecules **25–41** are tabulated in Table 1. In the physicochemical analysis, the values of all the molecules are displayed within the acceptable range, i.e., molecular weights, nHA (H-bond acceptors), nHD (H-bond donors), nRot (rotatable bonds), van der Waals volume, and TPSA (total polar surface area). The drug’s lipophilicity, which is essential for solubility, absorption, membrane penetration, plasma protein binding, distribution, and tissue penetration, is directly connected to the logP and logS values. The significance of the drug’s lipophilicity necessitated the inclusion of logP and logS as elements of Lipinski’s rule of five. In the present investigation, all these parameters were within the accepted range and displayed optimum oral bioavailability, indicating they can be developed to be delivered through the oral route [19,20].

The drug-like properties of the molecules are exemplified in Table 2. The different parameters, such as the QED (quantitative estimate of drug-likeness), NP score (natural product-likeness score), Lipinski rule, Pfizer rule, GSK rule, Golden Triangle rule, and Chelator rule, were calculated. The QED is an indicator of drug-likeness that was introduced in 2012 and is an index of drug-likeness that is modeled using the information available on marketed medications. It is frequently used in the present small-molecule drug development process for computational approaches and to assess drug-like features. Most of the designed compounds showed an attractive range of QED [21,22]. Typically, the NP score falls somewhere in the range of −5 to 5. If the score is higher, then there is a greater likelihood that the molecule in question is an NP [23,24]. All the designed molecules displayed NP-like properties except for **30**, which showed a −9.932 NP score. The compounds satisfying the GSK rule may have a more favorable ADMET profile, but unfortunately, only **25** accepted the rule. The compounds satisfying the Golden Triangle rule may have a more favorable ADMET profile, and all the molecules accepted under the rule show a favorable ADMET profile.

The absorption parameters of the molecules are illustrated in Table 3. As a model of how medications are absorbed by the human digestive tract, the human colon epithelial cancer cell line, known as Caco-2, is employed. This model is useful for determining whether a substance is appropriate for oral administration, predicting intestinal permeability, and researching drug efflux. Caco-2 permeability is optimum when the value is higher than −5.15 log units, and fortunately, all the molecules displayed optimum Caco-2 permeability [25]. It is possible to acquire a better knowledge of the process of drug efflux with the aid of MDCK-MDR1 cells, which also draws attention to early potential problems with drug permeability. It has been discovered that the permeability of MDCK-MDR1 may, in addition to intestinal permeability, be used as an accurate predictor of the permeability of the blood–brain barrier [26]. Many of the molecules displayed P-gp-inhibitor and P-gp-substrate activity. All the designed molecules displayed excellent human intestinal absorption (HIA). The molecules’ bioavailability of 20% and 30% were within acceptable limits.

The distribution and metabolism profile of the molecules are depicted in Table 4. The plasma protein binding (PPB, <90%) drugs with high protein bound within them may have a low therapeutic index. Many of the molecules displayed a PPB at less than 90%. The volume distribution (VD; optimal 0.04–20 L/kg) of all molecules was within the acceptable limit range. None of the molecules displayed a BBB penetration potential. Cytochrome (CYP) enzymes play an important role in drug metabolism; therefore, their substrate or inhibitor contributes to the drug’s action. None of the molecules in the current study demonstrated CYP inhibitory or substrate potential [27].

**Figure 2:**
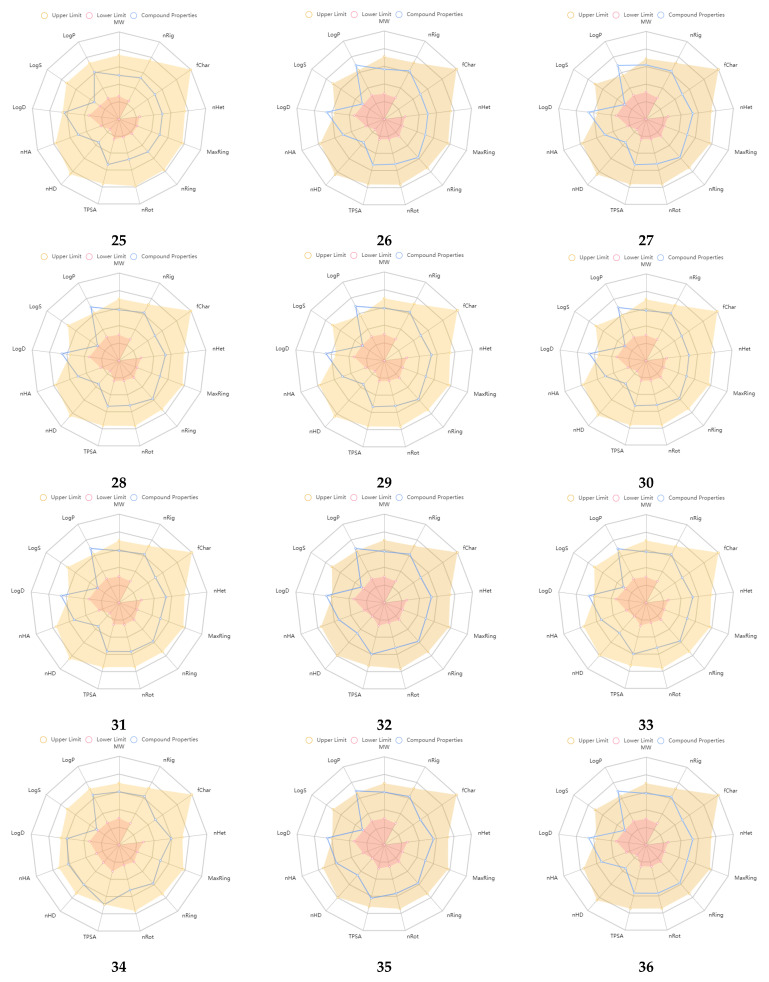

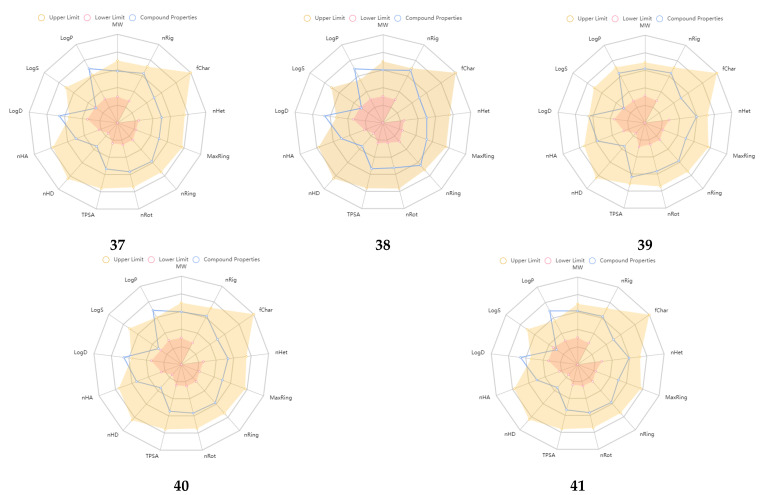
Physicochemical radar of developed molecules obtained from ADMETlab 2.0 web server.

An excretion and toxicity profile of the molecules is tabulated in Table 5. Many of the molecules displayed a moderate to low clearance (CL, High: >15 mL/min/kg; moderate: 5–15 mL/min/kg; low: <5 mL/min/kg) rate. All the molecules exhibited a short half-life (T_1/2_, <3 h). The toxicity profile of the molecules suggested favorable properties, and many of the values were within the range. The physicochemical radar of the developed molecules obtained from the ADMETlab 2.0 web server is reported in Figure 2, which indicates the molecules’ favorable physicochemical parameters to be developed further [27]. Most of the developed molecules displayed physicochemical properties within the upper limit of the acceptable range, as per the radar images. The physicochemical radar contains almost all the properties that are ideal for the development of any lead as a potential therapeutic agent. An environmental toxicity profile (bioconcentration factors, IGC_50_, LC_50_FM, and LC_50_DM) of the designed molecules is shown in Table 6. The environmental toxicity profile of the molecules was optimum and within the acceptable range.

### 2.2. Molecular Docking

In the docking calculations, comparisons have been made between the binding affinities of the designed derivatives and the binding mode of the native ligand that is found in the crystal structure of DHFR (PDB ID: 5CCC). The molecular interactions of the titled compounds are exemplified in the Supplementary Information; in Table 7, the most potent compounds’ 2D- and 3D-docking poses are described. The native ligand displayed a binding affinity with DHFR of −8.5 kcal/mol, and it established six conventional hydrogen bonds with Asp27, Ala6, Ile5, and Arg57, in addition to one carbon–hydrogen bond with Ile94. It has established many hydrophobic interactions, such as Pi–sigma bonds, Pi–Pi T-shaped bonds, alkyl bonds, and Pi–alkyl bonds with Ile50, Phe31, Ile94, Ile5, and Ala7. Compound **27** exhibited a binding affinity value of -8.6 kcal/mol with the formation of five hydrophobic bonds (pi-sigma, alkyl, pi-alkyl) with Leu28, Lys32, Leu28, Ala7, and Phe31. Compound **29** exhibited a binding affinity value of −9.3 kcal/mol with the formation of one hydrogen bond and several hydrophobic bonds (pi–sigma, pi–pi T-shaped alkyl, pi–alkyl) with Leu28, Phe31, Ile50, Ile5, Ala7, Met20, Trp30, and Phe31. Compound **30** displayed a binding affinity value of −9.6 kcal/mol with the formation of one carbon–hydrogen bond and many hydrophobic bonds (pi–sigma, pi–pi T-shaped alkyl, pi–alkyl) with Leu28, Phe31, Ile50, Ile5, Ala7, Met20, Trp30, and Phe31.

Compound **33** displayed a binding affinity value of −9.0 kcal/mol with the formation of two conventional hydrogen bonds and one carbon–hydrogen bond with Met20, Ile94, and Asp27. It also displayed many hydrophobic bonds (pi–sigma, pi–pi T-shaped alkyl, pi–alkyl) with Leu28, Leu54 Phe31, Ile50, Ile5, Ala7, Met20, Trp30, and Phe31. Compound **37** displayed a binding affinity value of −8.7 kcal/mol with the formation of one conventional hydrogen bond and one carbon–hydrogen bond with Ala7 and Asp27. It also displayed many hydrophobic bonds (pi–pi T-shaped, alkyl, pi–alkyl) with Tyr100, Ile50, Met20, Leu28, and Ile14. Compound **38** displayed a binding affinity value of −9 kcal/mol with the formation of one conventional hydrogen bond with Ile94. It also displayed many hydrophobic bonds (pi–pi T-shaped, amide-Pi stacked, alkyl, pi–alkyl) with Leu28, Met20, Ile5, Phe31, Ala7, and Phe31. It also displayed electrostatic interactions with Glu17. Compound **41** displayed a binding affinity value of −9 kcal/mol with the formation of one conventional hydrogen bond and one carbon bond with Thr113 and Trp30. It also displayed many hydrophobic bonds (pi–sigma, alkyl, pi–alkyl) with Leu28, Lys32, Ile5, Phe31, Ala7, and Phe31. Therefore, from the above results, the compounds that showed a binding affinity value lower than the native compound in the X-ray complex (**27, 29, 30, 33, 37, 38**, and **41**) were selected for the synthesis and biological evaluation.

### 2.3. Synthesis of the Selected Compounds

Compounds **27, 29, 30, 33, 37, 38**, and **41** were chosen for the synthesis based on the results of the in silico screening and molecular docking investigations.

**Figure 3:**
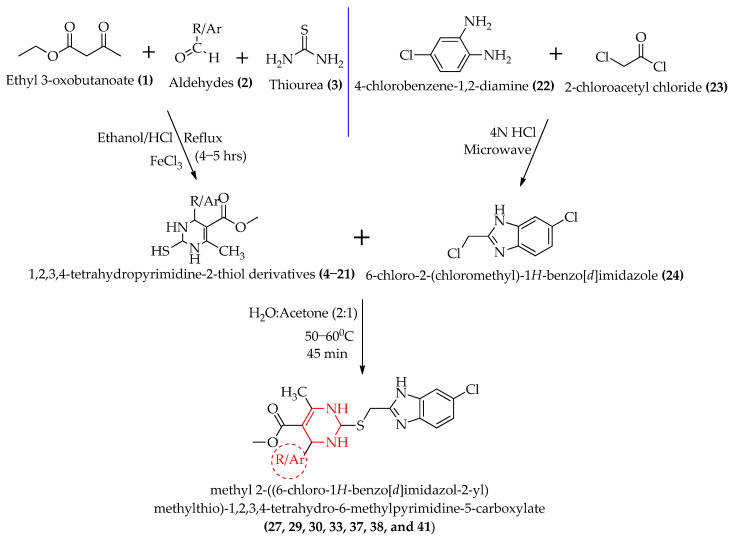
The reaction scheme for the synthesis of methyl 2-((6-chloro-1*H*-benzo[*d*]imidazol-2-yl)methylthio)-1,2,3,4-tetrahydro-6-methylpyrimidine-5-carboxylate derivatives.

Target derivatives can be prepared by condensing the suitable building blocks **4–21** with benzimidazole **24**. Initially, the 1,2,3,4-tetrahydropyrimidine-2-thiol derivatives **4–21** were synthesized using a modified Biginelli reaction, where a solution of ethyl acetoacetate **1**, (1.3 g, 10 mmol), thiourea **3**, (1.14 g, 15 mmol), ferric chloride (FeCl_3_.6H_2_O, 2.5 mmol), and conc. HCl (1–2 drops) in EtOH (20 mL) was heated with the appropriate aldehydes **2** (10 mmol) under reflux for 4–5 hrs. The yields obtained were in the range of 80–95%. Moreso, 6-Chloro-2-(chloromethyl)-1*H*-benzo[d]imidazole **24** was synthesized by a microwave-assisted synthesis by refluxing 4-chloro-1,2-phenylenediamine **22** and chloroacetyl chloride **23** in the presence of 4N HCl as the catalyst. The yield was 87%. The yellowish-brown product was recrystallized from dioxane; m.p. 142–144 °C. The detailed procedure of the synthesis and physical characterizations of compounds **4–21** and **24** are described in our previously published paper [28]. In the key step, **24** (1.66 g, 0.01 mol) and **4–21** (0.01 mol) were condensed by heating them with potassium hydroxide (KOH) in H_2_O: acetone (2:1) at about 50–60 °C for 45 min. The reaction mixture was cooled to room temperature and poured into ice-cold water; the precipitate was separated by filtration, and the products [methyl 2-((6-chloro-1*H*-benzo[*d*]imidazol-2-yl)methylthio)-1,2,3,4-tetrahydro-6-methylpyrimidine-5-carboxylate derivatives] **27, 29, 30, 33, 37, 38**, and **41** were recrystallized from ethanol. The yield was 80–90%. The reaction scheme for the synthesis is depicted in Figure 3.

### 2.4. In Vitro Antibacterial Activity

The findings of the synthetic derivatives’ antibacterial and antifungal activities are listed in Table 8, which display the MICs and MFCs, respectively (*n* = 3).

Xue-Qian Bai et al. reported some pyrimidine derivatives as potential antimicrobial agents, where one compound presented the most potent inhibitory activities against *Staphylococcus aureus*, *Escherichia coli*, and *Candida albicans*, with a MIC of 2.4 μmol/L. Additionally, it was the most potent, with MICs of 2.4 or 4.8 μmol/L against four multidrug-resistant, Gram-positive bacterial strains [29]. Omaima G. Shaaban et al. synthesized and evaluated some 3,4-dihydrothieno[2,3-d]pyrimidine derivatives as potential antimicrobial agents. Many of the derivatives displayed half of the potency of levofloxacin against *Pseudomonas aeruginosa* and *Proteus vulgaris* and also half the activity of ampicillin against the Gram-positive bacterium *B. subtilis* [30]. In the current study, each of the selected and synthetized compounds was tested for its in vitro antibacterial and antifungal activities using a variety of strains. All the compounds produced showed antibacterial activities against Gram-positive bacteria (*Staphylococcus aureus* and *Staphylococcus pyogenes*) as well as Gram-negative bacteria (*Escherichia coli* and *Pseudomonas aeruginosa*). All the compounds had actions against Gram-positive and Gram-negative bacteria that were much more powerful than that of ampicillin. Most of the compounds either had a higher potency than chloramphenicol or an equivalent potency to ciprofloxacin. Against *Escherichia coli*, **33** and **41** were sensitive at 25 µg/mL, whereas **29**, **37**, and **38** were sensitive at 50 µg/mL. It was observed that **27** and **30** were non-sensitive against *Escherichia coli*.

Infections brought on by the opportunistic bacteria *Pseudomonas aeruginosa* are often treated with the drug ciprofloxacin. In spite of the widespread administration of ciprofloxacin, the number of *Pseudomonas aeruginosa* strains that have developed resistance to the drug continue to rise [31]. Infections caused by *Pseudomonas aeruginosa* are notoriously difficult to treat because of the bacteria’s high levels of inherent and acquired antibiotic resistance. Once Pseudomonas aeruginosa has taken hold in a human host, it quickly creates genetic changes that make it resistant to antibiotics and better able to adapt to the host environment [32]. Therefore, it is not surprising that ciprofloxacin displayed low sensitivity (25 µg/mL) against *Pseudomonas aeruginosa*. However, all the compounds were sensitive at 50 µg/mL except **38**, which was non-sensitive against *Pseudomonas aeruginosa*. Against *Staphylococcus aureus*, **27**, **33**, **37**, **38**, and **41** were sensitive at 25 µg/mL, whereas **29** and **30** were sensitive at 50 µg/mL. Against *Staphylococcus pyogenes*, **30** and **33** were sensitive at 25 µg/mL, whereas **29**, **37**, **38**, and **41** were sensitive at 50 µg/mL. Compound **27** was non-sensitive against *Staphylococcus pyogenes*.

*Candida*, a yeast, is developing increased resistance to antifungal medications. *Candida* infections may be challenging to treat because of the possibility of drug resistance. Two patients with oral candidiasis, who did not improve while using nystatin in conjunction with triamcinolone acetonide, are presented. High in vitro resistance to nystatin was seen when triamcinolone acetonide was used in conjunction with the *Candida albicans* isolates collected from the patients after therapy [33]. This might be a reason why nystatin demonstrated low sensitivity (100 µg/mL) against *Candida albicans*, whereas all the compounds were sensitive at 100 µg/mL, which is equipotent to nystatin and more potent than griseofulvin except for 33, which was sensitive at 200 µg/mL. Against Aspergillus niger, all the compounds were equipotent with nystatin and griseofulvin (100 µg/mL) except for **30**, which was sensitive at 200 µg/mL and **37** was non-sensitive. Against *Aspergillus clavatus*, all the compounds were sensitive at 100 µg/mL except for **38** and **41**, which were non-sensitive. It was observed that **29** and **33** were sensitive against all the bacterial and fungal strains.

From the above results, it was observed that these molecules have enough potential as antimicrobial agents. These molecules displayed an optimum binding affinity for the DHFR enzyme and showed significant inhibition. Therefore, we proposed that these molecules exert antimicrobial activity via the inhibition of DHFR.

## 3. Materials and Methods

### 3.1. Pre-ADMET Profile and Drug-Likeness Properties

The in silico ADMET assessment models are a new type of tool that has been created to provide medicinal chemists with extra support in the process of the creation and optimization of leads. ADMETlab 2.0 is a revamped version of the AMDETlab web server, which is commonly used for predicting the pharmacokinetics and toxic characteristics of various compounds (https://admetmesh.scbdd.com/ accessed on 21 August 2022) [27].

### 3.2. Molecular Docking

**Figure 4:**
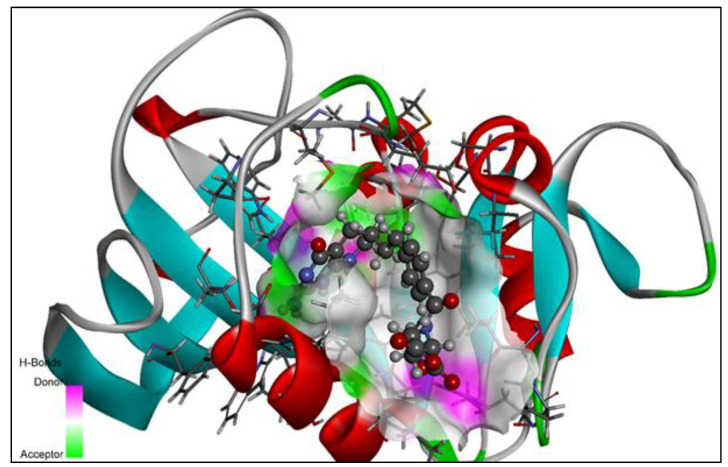
The native ligand 5,10-dideazatetrahydrofolic acid is shown in the allosteric location of the DHFR in this 3D ribbon image.

Using Autodock vina in PyRx 0.8, the hypothesized derivatives, as well as the native ligand, were docked to the crystal structure of the wild-type *E. coli* dihydrofolate reductase [34]. The structures of the proposed derivatives and the native ligand were drawn using ChemDraw Ultra 12.0. (Mol File format). By using the open-Babel tool, the ligands were imported into the PyRx software. By using the Universal Force Field (UFF), each of the ligands was optimized in terms of reducing the amount of energy [35]. The ligands were then converted to the PDBQT format and prepared for docking purposes. The crystal structure of wild-type *E. coli* dihydrofolate reductase was obtained from the RCSB Protein Data Bank (PDB) with PDB ID: 5CCC (https://www.rcsb.org/structure/5CCC, accessed on 28 August 2022). The enzyme structure was refined using Discovery Studio Visualizer (version 19.1.0.18287), and then it was purified and prepared for docking using the same program [36]. The output file of the enzyme was saved in a PDB file format and imported to PyRx to perform the molecular docking studies. In order to aid molecular docking, a three-dimensional grid box (size_x = 41.7862652138Å; size_y = 39.1754565902Å; size_z = 37.1398050256Å) with an exhaustiveness value of eight was developed [34]. The strategy reported in previous papers was used in order to carry out the complete molecular docking method as well as to locate cavities and active amino acid residues [18,28,37,38,39,40,41,42,43,44,45,46]. The exposed cavity of the DHFR is shown with the co-crystallized ligand molecule in Figure 4.

### 3.3. Chemistry

From the Lab Trading Laboratory in Aurangabad, Maharashtra, India, all the essential chemicals and reagents of synthetic quality were obtained. The progression of the reaction was monitored and verified using thin-layer chromatography (TLC, Merck precoated silica GF 254). The melting points were determined using a VEEGO Model VMP-D melting point apparatus. The mass spectra were determined by SAPALA ORGANICS PVT LTD using an LC-MS spectrometer, and the results are reported in the Supplementary Information. ^1^H NMR and ^13^C NMR were calculated. CDCl_3_ was used as the solvent, and TMS was used as the internal standard. The chemical shift values were stated in δ ppm.

*Methyl 2-((6-chloro-1H-benzo[d]imidazol-2-yl)methylthio)-4-(4-bromophenyl)-1,2,3,4-tetrahydro-6-methylpyrimidine-5-carboxylate* **(27)**

Molecular formula: C_21_H_20_BrClN_4_O_2_S, molecular weight: 508.50 g/mol; m.p. (^0^C): 208–210; R_f_ Value: 0.85; % Yield: 78. Solubility: ethanol, methanol, DCM, chloroform. Elemental analysis (*calc.*): C, 49.67; H, 3.97; Br, 15.73; Cl, 6.98; N, 11.03; S, 6.31. ^1^H NMR δ ppm: 1.72 (*s*, pyrimidine 6-methyl protons, 3H), 2.01, 2.12 (two-*s*, pyrimidine ―NH, 2H), 3.72 (s, ―SCH_2_, 2H), 3.77 (*s*, acetatemethyl, 3H), 4.60 (*s*, 4H-pyrimidine, 1H), 4.81 (*s*, 2H-pyrimidine, 1H), 5.05 (*s*, imidazole-NH, 1H), 7.12, 7.85 (*q*, Ar-H, 4H, J = 1.5 and 7.5), 7.14, 7.53, 8.36 (*t*, Ar-H, 3H, J = 7.5). ^13^C NMR δ ppm: 14.1 (C10), 26.3 (C19), 52.3 (C30), 58.9 (C3), 81.4 (C1), 106 (C4), 115.8 (C28), 116.6 (C25), 121.4 (C14), 124.1 (C26), 129.2 (C27), 130.1 (C9 and C12), 131.4 (C13 and C15), 137 (C23), 138.5 (C11), 140.3 (C22), 141.5 (C20), 153.9 (C5), 167.2 (C7). MS: m/z 508.34, 509.32 (m + 1), 510.90 (m + 2).

*Methyl 2-((6-chloro-1H-benzo[d]imidazol-2-yl) methylthio)-4-(4-chlorophenyl)-1, 2, 3, 4-tetrahydro-6-methylpyrimidine-5-carboxylate* **(29)**

Molecular formula: C_21_H_20_Cl_2_N_4_O_2_S, molecular weight: 464.38 gm/mol; m.p. (°C): 213–216; R_f_ Value: 0.85; % Yield: 76. Solubility: ethanol, methanol, DCM, chloroform. Elemental analysis (*calc.*): C, 54.43; H, 4.35; Cl, 15.30; N, 12.09; S, 6.92. ^1^H NMR δ ppm: 1.91, 2.0 (two-*s*, pyrimidine ―NH, 2H), 3.26 (*s*, pyrimidine 6-methyl protons, 3H), 3.70 (*s*, ―SCH_2_, 2H), 3.77 (*s*, acetatemethyl, 3H), 4.58 (*s*, 4H-pyrimidine, 1H), 4.80 (*s*, 2H-pyrimidine, 1H), 5.0 (*s*, imidazole-NH, 1H), 7.34, 7.37 (*q*, Ar-H, 4H, J = 1.5 and 7.5), 7.14, 7.53, 8.36 (*t*, Ar-H, 3H, J = 7.5). ^13^C NMR δ ppm: 14.1 (C10), 26.3 (C19), 52.3 (C30), 58.9 (C3), 81.4 (C1), 106 (C4), 115.8 (C28), 116.6 (C25), 121.4 (C14), 124.1 (C26), 129.2 (C27), 130.1 (C9 and C12), 131.4 (C13 and C15), 137 (C23), 138.5 (C11), 140.3 (C22), 141.5 (C20), 153.9 (C5), 167.2 (C7). MS: m/z 464.16, 465.17 (m + 1), 466.17 (m + 2).

*Methyl 2-((6-chloro-1H-benzo[d]imidazol-2-yl)methylthio)-1,2,3,4-tetrahydro-6-methyl- 4-p-tolylpyrimidine-5-carboxylate* **(30)**

Molecular formula: C_22_H_23_ClN_4_O_2_S, molecular weight: 443.22 g/mol; m.p. (°C): 228–238; R_f_ Value: 0.59; % Yield: 50. Solubility: ethanol, methanol, DCM, chloroform. Elemental analysis (*calc.*): C, 59.65; H, 5.23; Cl, 8.00; N, 12.65; S, 7.24. ^1^H NMR δ ppm: 1.91, 2.0 (two-*s*, pyrimidine ―NH, 2H), 2.26 (*s*, pyrimidine 6-methyl protons, 3H), 2.34 (*s*, 4-methyl of phenyl, 3H), 3.70 (*s*, ―SCH_2_, 2H), 3.77 (*s*, acetatemethyl, 3H), 4.59 (*s*, 4H-pyrimidine, 1H), 4.80 (*s*, 2H-pyrimidine, 1H), 5.0 (*s*, imidazole-NH, 1H), 7.11 (*q*, Ar-H, 4H, J = 7.5), 7.14, 7.53, 8.36 (t, Ar-H, 3H, J = 7.5). ^13^C NMR δ ppm: 14.1 (C17), 21.3 (C16), 26.3 (C19), 52.3 (C30), 58.9 (C3), 81.4 (C1), 106 (C4), 115.8 (C28), 116.6 (C25), 124.1 (C26), 127.8 (C9 and C11), 128.8 (C14 and C15), 129.2 (C27), 136.5 (C10), 136.7 (C12), 137 (C23), 140.3 (C22), 141.5 (C20), 153.9 (C5), 167.2 (C7). MS: m/z 431.15, 432.15 (m + 1), 433.16 (m + 2).

*Methyl 2-((6-chloro-1H-benzo[d]imidazol-2-yl)methylthio)-1,2,3,4-tetrahydro-4-(3-hy-droxyphenyl)-6-methylpyrimidine-5-carboxylate* **(33)**

Molecular formula: C_22_H_23_ClN_4_O_2_S, molecular weight: 445.42 g/mol; m.p. (°C): 268–286; R_f_ Value: 0.68; % Yield: 50. Solubility: ethanol, methanol, DCM, chloroform. Elemental analysis (*calc.*): C, 57.69; H, 4.74; Cl, 7.93; N, 12.60; O, 10.79; S, 7.21. ^1^H NMR δ ppm: 1.91, 2.0 (two-*s*, pyrimidine ―NH, 2H), 2.26 (*s*, pyrimidine 6-methyl protons, 3H), 3.70 (*s*, ―SCH_2_, 2H), 3.77 (*s*, acetatemethyl, 3H), 4.59 (s, 4H-pyrimidine, 1H), 4.80 (*s*, 2H-pyrimidine, 1H), 5.0 (*s*, imidazole-NH, 1H), 5.35 (*s*, ―OH, 1H), 6.76, 6.79, 6.97, 7.16 (*q*, Ar-H, 4H, J = 1.5 and 7.5), 7.14, 7.53, 8.36 (t, Ar-H, 3H, J = 7.5). ^13^C NMR δ ppm: 14.1 (C10), 26.3 (C19), 52.3 (C30), 59.2 (C3), 81.4 (C1), 106 (C4), 113.6 (C12), 114.2 (C14), 115.8 (C28), 116.6 (C25), 120.5 (C16), 124.1 (C26), 129.2 (C27), 129.9 (C15), 137 (C23), 139.5 (C11), 140.3 (C22), 141.5 (C20), 153.9 (C5), 156.8 (C13), 167.2 (C7). MS: m/z 445.13, 446.14 (m + 1), 447.14 (m + 2).

*Methyl 2-((6-chloro-1H-benzo[d]imidazol-2-yl)methylthio)-1,2,3,4-tetrahydro-6-methyl-4-styrylpyrimidine-5-carboxylate* **(37)**

Molecular formula: C_23_H_23_ClN_4_O_2_S, molecular weight: 455.97 gm/mol; m.p. (°C): 280–295; R_f_ Value: 0.90; % Yield: 78. Solubility: ethanol, methanol, DCM, chloroform. Elemental analysis (*calc.*): C, 60.72; H, 5.10; Cl, 7.79; N, 12.31; S, 7.05. ^1^H NMR δ ppm: 1.91, 2.0 (two-*s*, pyrimidine ―NH, 2H), 2.26 (*s*, pyrimidine 6-methyl protons, 3H), 3.70 (*s*, ―SCH_2_, 2H), 3.77 (*s*, acetatemethyl, 3H), 3.99 (*s*, 4H-pyrimidine, 1H), 4.80 (*s*, 2H-pyrimidine, 1H), 5.0 (*s*, imidazole-NH, 1H), 6.19, 6.56 (d, ethelene protons, 2H, J = 15.1), 7.24, 7.40, 7.33 (t, Ar-H, 5H, J = 7.5), 7.14, 7.53, 8.36 (t, Ar-H, 3H, J = 7.5). ^13^C NMR δ ppm: 14.2 (C18), 26.3 (C20), 51.0 (C3), 52.3 (C31), 81.9 (C1), 106.0 (C4), 115.8 (C29), 116.6 (C26), 123.3 (C10), 124.1 (C27), 127.9 (C15), 128.5 (C13 and C17), 128.6 (C14 and C16), 129.2 (C28), 136.4 (C12), 137 (C24), 140.3 (C23), 141.5 (C21), 153.9 (C5), 167.2 (C7). MS: m/z 455.13, 456.14 (m + 1), 457.14 (m + 2).

*Methyl 2-((6-chloro-1H-benzo[d]imidazol-2-yl)methylthio)-1,2,3,4-tetrahydro-6-methyl-4-(naphthalen-1-yl)pyrimidine-5-carboxylate* **(38)**

Molecular formula: C_25_H_23_ClN_4_O_2_S, molecular weight: 479 g/mol; m.p. (°C): 280–295; R_f_ Value: 0.90; % Yield: 70. Solubility: ethanol, methanol, DCM, chloroform. Elemental analysis (*calc.*): C, 62.69; H, 4.84; Cl, 7.40; N, 11.70; S, 6.69. ^1^H NMR δ ppm: 1.91, 2.0 (two-*s*, pyrimidine ―NH, 2H), 2.26 (*s*, pyrimidine 6-methyl protons, 3H), 3.70 (*s*, ―SCH_2_, 2H), 3.77 (*s*, acetatemethyl, 3H), 4.59 (*s*, 4H-pyrimidine, 1H), 4.80 (*s*, 2H-pyrimidine, 1H), 5.0 (*s*, imidazole-NH, 1H), 7.00, 7.42, 7.52, 7.54, 7.92, 8.05, 8.18 (m, napthyl-H, 7H, J = 1.5 and 7.5), 7.14, 7.53, 8.36 (t, Ar-H, 3H, J = 7.5). ^13^C NMR δ ppm: 14.1 (C20), 26.3 (C22), 52.3 (C33), 57.2 (C3), 81.4 (C1), 106.0 (C4), 115.8 (C31), 116.6 (C28), 124.1 (C29), 124.2 (C11), 125.5 (C18), 125.6 (C16), 125.8 (C17), 126.5 (C13), 126.9 (C12), 132.6 (C19), 133.5 (C14), 134.0 (C10), 137.0 (C26), 140.3 (C25), 141.5 (C23), 153.9 (C5), 167.2 (C7). MS: m/z 479.13, 480.14 (m + 1), 481.14 (m + 2).

*Methyl 2-((6-chloro-1H-benzo[d]imidazol-2-yl)methylthio)-4-(4-(trifluoromethyl) phenyl)-1,2,3,4-tetrahydro-6-methylpyrimidine-5-carboxylate* **(41)**

Molecular formula: C_22_H_20_ClF_3_N_4_O_2_S, molecular weight: 497.90 m/mol; m.p. (°C): 259–278; R_f_ Value: 0.89; % Yield: 76. Solubility: ethanol, methanol, DCM, chloroform. Elemental analysis (*calc.*): C, 53.17; H, 4.06; Cl, 7.13; F, 11.457; N, 11.27; S, 6.45. ^1^H NMR δ ppm: 1.91, 2.0 (two-*s*, pyrimidine ―NH, 2H), 2.26 (*s*, pyrimidine 6-methyl protons, 3H), 3.70 (*s*, ―SCH_2_, 2H), 3.77 (*s*, acetatemethyl, 3H), 4.59 (*s*, 4H-pyrimidine, 1H), 4.80 (*s*, 2H-pyrimidine, 1H), 5.0 (*s*, imidazole-NH, 1H), 7.16, 7.50 (d, Ar-H, 4H, J = 7.5), 7.14, 7.53, 8.36 (t, Ar-H, 3H, J = 7.5). ^13^C NMR δ ppm: 14.1 (C10), 26.3 (C22), 52.3 (C33), 58.9 (C3), 81.4 (C1), 106.0 (C4), 115.8 (C31), 116.6 (C28), 124.1 (C29 and C17), 124.9 (C13 and C15), 128.2 (C9 and C12), 129.2 (C30), 129.3 (C14), 137.0 (C26), 140.3 (C25), 141.5 (C23), 153.9 (C5), 167.2 (C7). MS: m/z 497.13, 498.14 (m + 1), 499.14 (m + 2).

### 3.4. In Vitro Biological Evaluation

By using the broth dilution method, several different doses of derivatives were produced in the DMSO so that their antibacterial and antifungal properties could be evaluated against the reference strains (Gram-positive bacteria (*Staphylococcus aureus* and *Staphylococcus pyogenes*), Gram-negative bacteria (*Escherichia coli* and *Pseudomonas aeruginosa*), and fungal (*Candida albicans*, *Aspergillus niger*, and *Aspergillus clavatus*)). The bacteria were kept alive in a nutrient-rich Mueller–Hinton broth, and the drugs were diluted. The turbidity of the broth was used to identify the test strains that were used to inoculate the broth with 10^8^ colony-forming units (cfu) per milliliter. For both the primary and secondary screenings, stock solutions of the synthesized derivate were diluted in a step-by-step process to a concentration of 2 mg/mL. The first screen comprised screening of the synthesized derivatives at concentrations of 1000, 500, and 250 μg/mL; further screenings of the active derivatives were performed at concentrations of 200, 100, 50, 25, 12.5, and 6.250 μg/mL. A control that did not contain antibiotics was subcultured (before being inoculated) by distributing one loopful of media uniformly over a fourth of a plate of medium that was adequate for growing the test organisms. This was followed by overnight incubation at 37 °C. The lowest concentrations of derivatives that were able to prevent the development of bacteria or fungi were used as the minimal inhibitory concentrations (MICs). In order to establish the correctness of the MIC, it was compared with the quantity of control growth that occurred before the incubation process (the original inoculum). The antibiotics gentamycin, ampicillin, chloramphenicol, ciprofloxacin, and norfloxacin served as the standards for determining the antibacterial activity, while nystatin and griseofulvin served as the criteria for determining the antifungal activity [28,40,46].

## 4. Conclusions

We designed and developed some methyl 2-((6-chloro-1*H*-benzo[*d*]imidazol-2-yl)methylthio)-1,2,3,4-tetrahydro-6-methylpyrimidine-5-carboxylate derivatives, **25–41**, as potential DHFR inhibitors. The ADMET profiles of all the created compounds were positive, and some of them even showed reduced binding affinities (in terms of DHFR) compared to the native ligand. In the present investigation, several bacterial and fungal strains (Gram-positive bacteria (*Staphylococcus aureus* and *Staphylococcus pyogenes*), Gram-negative bacteria (*Escherichia coli* and *Pseudomonas aeruginosa*), and fungal (*Candida albicans*, *Aspergillus niger*, and *Aspergillus clavatus*)) were used to evaluate the in vitro antibacterial and antifungal properties of the synthesized compounds. It was observed that **29** and **33** were sensitive against all the bacterial and fungal strains. The results showed that the most promising compounds were those derived from 1,2,3,4-tetrahydropyrimidine-2-thiol with a 6-chloro-2-(chloromethyl)-1*H*-benzo[*d*]imidazole moiety, possibly because this moiety plays a vital role in boosting the compounds’ antibacterial characteristics. These compounds inhibited the DHFR enzyme with substantial binding affinity. Thus, we reasoned, these compounds may be antimicrobial because they inhibit DHFR. We thus conclude that these compounds have the potential to serve as lead compounds for the creation of further effective antibacterial and antifungal compounds.

## Figures and Tables

**Table 1:** Physicochemical properties of methyl 2-((6-chloro-1*H*-benzo[*d*]imidazol-2-yl)methylthio)-1,2,3,4-tetrahydro-6-methylpyrimidine-5-carboxylate derivatives (**25**–**41**).

Code	Physicochemical Properties							
	Molecular Weight	Volume	nHA	nHD	nRot	TPSA	logS	logP
**25**	352.08	321.796	6	2	5	79.37	−3.259	2.224
**26**	428.11	409.106	6	2	6	79.37	−3.862	3.626
**27**	506.02	428.389	6	2	6	79.37	−4.092	4.499
**28**	446.1	415.173	6	2	6	79.37	−3.932	3.76
**29**	462.07	424.317	6	2	6	79.37	−4.108	4.333
**30**	442.12	426.402	6	2	6	79.37	−4.008	4.038
**31**	458.12	435.192	7	2	7	88.6	−4.046	3.705
**32**	444.1	417.896	7	3	6	99.6	−3.713	3.247
**33**	444.1	417.896	7	3	6	99.6	−3.713	3.27
**34**	476.09	435.476	9	5	6	140.06	3.766	2.452
**35**	474.11	443.982	8	3	7	108.83	−3.859	3.201
**36**	458.12	435.192	7	2	7	88.6	−4.027	3.56
**37**	454.12	441.061	6	2	7	79.37	−4.143	3.796
**38**	478.12	464.46	6	2	6	79.37	−4.21	4.716
**39**	506.08	462.491	8	2	7	113.51	−4.064	2.474
**40**	471.15	454.695	7	2	7	82.61	−3.681	3.891
**41**	496.09	444.604	6	2	7	79.37	−4.57	4.465

**Table 2:** Drug-likeness properties of methyl 2-((6-chloro-1*H*-benzo[*d*]imidazol-2-yl)methylthio)-1,2,3,4-tetrahydro-6-methylpyrimidine-5-carboxylate derivatives (**25**–**41**).

Code	Medicinal Chemistry						
	QED	NP score	Lipinski Rule	Pfizer Rule	GSK Rule	Golden Triangle	Chelator Rule
**25**	0.827	−1.057	Accepted	Accepted	Accepted	Accepted	0 alerts
**26**	0.591	−0.895	Accepted	Accepted	Rejected	Accepted	0 alerts
**27**	0.47	−0.943	Accepted	Accepted	Rejected	Rejected	0 alerts
**28**	0565	−1.108	Accepted	Accepted	Rejected	Accepted	0 alerts
**29**	0.522	−0.861	Accepted	Accepted	Rejected	Accepted	0 alerts
**30**	0.563	−9.932	Accepted	Accepted	Rejected	Accepted	0 alerts
**31**	0.534	−0.838	Accepted	Accepted	Rejected	Accepted	0 alerts
**32**	0.514	−0.637	Accepted	Accepted	Rejected	Accepted	0 alerts
**33**	0.514	−0.692	Accepted	Accepted	Rejected	Accepted	0 alerts
**34**	0.278	−0.358	Accepted	Accepted	Rejected	Accepted	1 alert
**35**	0.462	−0.53	Accepted	Accepted	Rejected	Accepted	1 alert
**36**	0.534	−0.863	Accepted	Accepted	Rejected	Accepted	0 alerts
**37**	0.529	−0.642	Accepted	Accepted	Rejected	Accepted	0 alerts
**38**	0.371	−0.872	Accepted	Accepted	Rejected	Accepted	0 alerts
**39**	0.489	−1.112	Accepted	Accepted	Rejected	Rejected	0 alerts
**40**	0.519	−1.024	Accepted	Accepted	Rejected	Accepted	0 alerts
**41**	0.466	−1.05	Accepted	Accepted	Rejected	Accepted	0 alerts

**Table 3:** An absorption parameter of methyl 2-((6-chloro-1*H*-benzo[*d*]imidazol-2-yl)methylthio)-1,2,3,4-tetrahydro-6-methylpyrimidine-5-carboxylate derivatives (**25**–**41**).

Code	Absorption						
	Caco-2 Permeability	MDCK Permeability	P-gp Inhibitor	P-gp Substrate	HIA	F20%	F30%
**25**	−5.323	6 × 10^−6^	0.001	0.44	0.016	0.001	0.009
**26**	−5.316	1.1 × 10^−5^	0.02	0.025	0.005	0.004	0.002
**27**	−5.162	1.4 × 10^−5^	0.37	0.094	0.053	0.002	0.002
**28**	−5.175	1.1 × 10^−5^	0.141	0.206	0.005	0.002	0.004
**29**	−5.155	1.3 × 10^−5^	0.06	0.115	0.005	0.002	0.002
**30**	−5.216	8 × 10^−6^	0.704	0.106	0.004	0.003	0.003
**31**	−5.337	7 × 10^−6^	0.126	0.152	0.005	0.002	0.011
**32**	−5.337	6 × 10^−6^	0.018	0.057	0.006	0.003	0.002
**33**	−5.613	6 × 10^−6^	0.025	0.033	0.006	0.003	0.002
**34**	−6.13	4 × 10^−6^	0.006	0.59	0.01	0.07	0.006
**35**	−5.593	5 × 10^−6^	0.028	0.574	0.009	0.004	0.009
**36**	−5.315	8 × 10^−6^	0.074	0.246	0.007	0.003	0.013
**37**	−5.238	1.2 × 10^−5^	0.028	0.02	0.006	0.002	0.003
**38**	−5.253	9 × 10^−6^	0.166	0.029	0.005	0.005	0.002
**39**	−5.898	9 × 10^−6^	0.087	0.199	0.005	0.002	0.002
**40**	−5.313	9 × 10^−6^	0.714	0.858	0.009	0.011	0.009
**41**	−5.302	2 × 10^−5^	0.615	0.062	0.004	0.002	0.002

**Table 4:** Distribution and metabolism profile of methyl 2-((6-chloro-1*H*-benzo[*d*]imidazol-2-yl)methylthio)-1,2,3,4-tetrahydro-6-methylpyrimidine-5-carboxylate derivatives (**25**–**41**).

Code	Distribution				Metabolism									
	PPB	VD	BBB Penetration	Fu	CYP1A2		CYP2C19		CYP2C9		CYP2D6		CYP3A4	
					Inhibitor	Substrate	Inhibitor	Substrate	Inhibitor	Substrate	Inhibitor	Substrate	Inhibitor	Substrate
**25**	54.15	1.805	0.727	40.82	0.924	0.963	0.971	0.53	0.257	0.117	0.512	0.104	0.798	0.266
**26**	87.83	1.31	0.821	10.01	0.917	0.952	0.984	0.175	0.94	0.089	0.92	0.103	0.951	0.661
**27**	94.22	1.442	0.835	6.893	0.935	0.946	0.978	0.228	0.954	0.131	0.947	0.113	0.962	0.696
**28**	90.64	1.202	0.805	8.954	0.929	0.952	0.978	0.195	0.944	0.133	0.923	0.118	0.955	0.627
**29**	94.94	1.043	0.726	5.139	0.942	0.957	0.982	0.179	0.954	0.103	0.939	0.101	0.961	0.815
**30**	91.91	1.068	0.789	8.289	0.918	0.945	0.98	0.489	0.949	0.174	0.935	0.143	0.961	0.815
**31**	89.30	1.21	0.484	7.920	0.921	0.949	0.981	0.585	0.949	0.391	0.937	0.178	0.967	0.678
**32**	87.66	1.289	0.605	10.02	0.874	0.951	0.981	0.223	0.927	0.414	0.898	0.117	0.939	0.339
**33**	86.59	1.332	0.642	9.720	0.905	0.952	0.982	0.134	0.928	0.229	0.909	0.11	0.954	0.335
**34**	96.31	0.881	0.07	3.163	0.475	0.95	0.94	0.067	0.889	0.464	0.739	0.13	0.873	0.268
**35**	92.83	1.142	0.488	5.388	0.887	0.965	0.981	0.495	0.921	0.553	0.856	0.154	0.953	0.578
**36**	89.41	1.133	0.581	8.664	0.938	0.964	0.986	0.677	0.933	0.241	0.090	0.122	0.956	0.833
**37**	92.11	1.185	0.783	10.49	0.959	0.878	0.984	0.126	0.943	0.108	0.936	0.11	0.958	0.431
**38**	96.01	0.756	0.565	4.051	0.952	0.957	0.981	0.152	0.956	0.177	0.962	0.129	0.969	0.805
**39**	79.91	1.28	0.793	18.03	0.637	0.969	0.965	0.571	0.879	0.106	0.819	0.085	0.939	0.814
**40**	88.55	1.513	0.877	9.325	0.933	0.95	0.979	0.689	0.942	0.098	0.879	0.175	0.956	0.614
**41**	95.31	2.268	0.489	5.676	0.889	0.962	0.97	0.169	0.958	0.155	0.931	0.112	0.962	0.607

**Table 5:** Excretion and toxicity profile of methyl 2-((6-chloro-1*H*-benzo[*d*]imidazol-2-yl)methylthio)-1,2,3,4-tetrahydro-6-methylpyrimidine-5-carboxylate derivatives (**25**–**41**).

Code	Excretion		Toxicity									
	CL	T1/2	H-HT	DILI	AMES Toxicity	Rat Oral Acute Toxicity	FDAMDD	Skin Sensitization	Carcinogenicity	Eye Corrosion	Eye Irritation	Respiratory Toxicity
**25**	5.157	0.749	0.701	0.824	0.022	0.92	0.932	0.294	0.171	0.003	0.011	0.912
**26**	4.619	0.207	0.808	0.96	0.417	0.939	0.951	0.118	0.077	0.003	0.008	0.895
**27**	3.126	0.109	0.498	0.956	0.041	0.967	0.957	0.117	0.11	0.003	0.007	0.845
**28**	4.672	0.104	0.878	0.951	0.591	0.956	0.968	0.098	0.095	0.003	0.007	0.863
**29**	4.404	0.124	0.763	0.958	0.359	0.952	0.955	0.106	0.078	0.003	0.007	0.836
**30**	4.578	0.162	0.792	0.957	0.438	0.954	0.953	0.107	0.086	0.003	0.007	0.88
**31**	5.029	0.187	0.817	0.953	0.767	0.94	0.949	0.103	0.062	0.003	0.007	0.879
**32**	4.884	0.541	0.749	0.954	0.177	0.872	0.933	0.118	0.094	0.003	0.007	0.852
**33**	5.058	0.55	0.175	0.945	0.122	0.845	0.954	0.123	0.081	0.003	0.007	0.871
**34**	4.12	0.818	0.556	0.97	0.09	0.22	0.891	0.421	0.085	0.003	0.008	0.875
**35**	5.168	0.585	0.779	0.943	0.168	0.757	0.941	0.102	0.067	0.003	0.007	0.886
**36**	5.004	0.321	0.77	0.949	0.493	0.939	0.947	0.095	0.072	0.003	0.007	0.899
**37**	4.314	0.281	0.862	0.959	0.703	0.956	0.949	0.121	0.065	0.003	0.008	0.895
**38**	4.459	0.133	0.878	0.966	0.893	0.964	0.962	0.143	0.17	0.003	0.008	0.882
**39**	3.046	0.102	0.957	0.989	0.023	0.829	0.954	0.068	0.127	0.003	0.005	0.651
**40**	4.899	0.203	0.871	0.959	0.917	0.952	0.93	0.137	0.194	0.003	0.007	0.96
**41**	4.747	0.05	0.921	0.952	0.032	0.985	0.959	0.075	0.072	0.003	0.007	0.921

**Table 6:** Environmental toxicity profile of methyl 2-((6-chloro-1*H*-benzo[*d*]imidazol-2-yl)methylthio)-1,2,3,4-tetrahydro-6-methylpyrimidine-5-carboxylate derivatives (**25**–**41**).

Code	Environmental Toxicity			
	Bio Concentration Factors	IGC_50_	LC_50_FM	LC_50_DM
**25**	0.112	3.994	3.84	5.635
**26**	0.397	4.215	4.622	5.922
**27**	0.806	4.769	5.632	6.517
**28**	0.785	4.481	4.887	6.707
**29**	0.772	4.69	5.246	6.315
**30**	0.434	4.408	4.853	6.068
**31**	0.534	4.531	5.089	6.357
**32**	0.31	4.681	4.735	6.019
**33**	0.309	4.642	4.663	5.986
**34**	0.375	4.644	4.621	5.93
**35**	0.449	4.694	4.821	6.267
**36**	0.505	4.455	4.909	6.144
**37**	1.052	4.641	6.572	6.504
**38**	0.813	4.852	5.588	6.329
**39**	0.053	3.525	3.946	5.536
**40**	0.373	4.621	4.956	6.331
**41**	1.059	4.615	5.523	6.622

**Table 7:** The 2D- and 3D-docking postures of molecules selected for the synthesis.

2D-Binding Orientations	3D-Binding Orientations
**Native Ligand**	
** 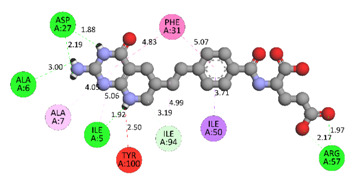 **	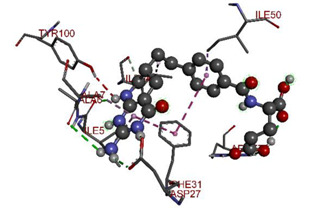
**27**	
** 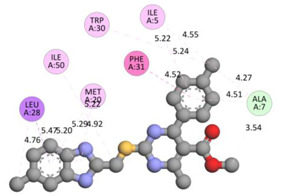 **	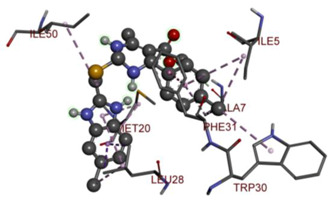
**29**	
** 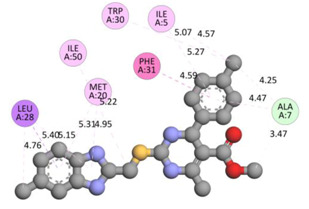 **	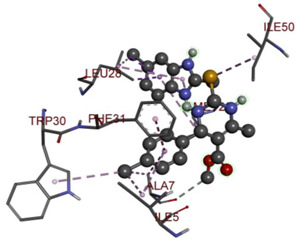
**30**	
** 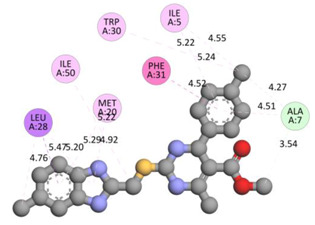 **	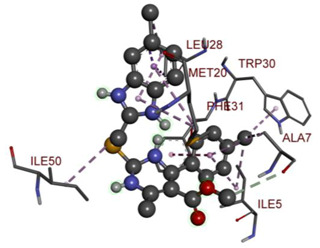
**33**	
** 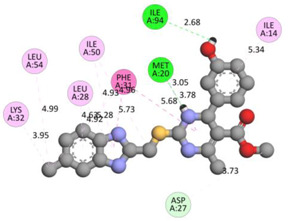 **	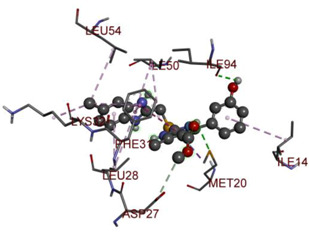
**37**	
** 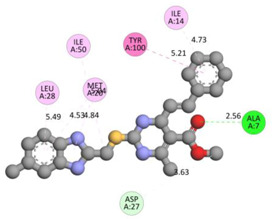 **	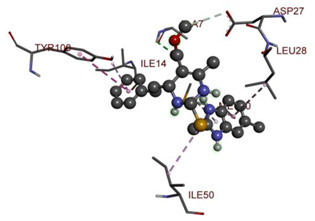
**38**	
** 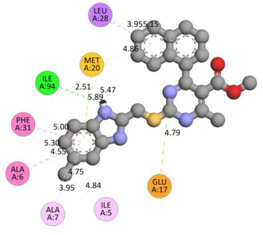 **	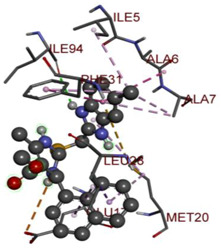
**41**	
** 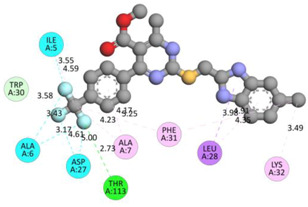 **	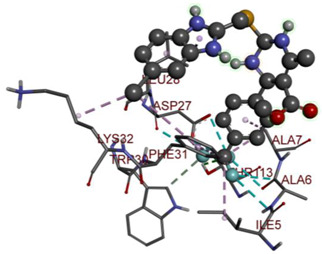

**Table 8:** The antibacterial and antifungal properties shown by the compounds that were synthesized (*n* = 3).

Compound Code	Antimicrobial Activity [*MIC* (µg/mL)]				Antifungal Activity [*MFC* (µg/mL)]		
	*E.C.*	*P.A.*	*S.A.*	*S.P.*	*C.A.*	*A.N.*	*A.C.*
**27**	NS	50	25	NS	100	100	100
**29**	50	50	50	50	100	100	100
**30**	NS	50	50	25	100	200	100
**33**	25	50	25	25	200	100	100
**37**	50	50	25	50	100	NS	100
**38**	50	NS	25	50	100	100	NS
**41**	25	50	25	50	100	100	NS
Gentamycin	0.05	1	0.25	0.5	NA	NA	NA
Ampicillin	100	NA	250	100	NA	NA	NA
Chloramphenicol	50	50	50	50	NA	NA	NA
Ciprofloxacin	25	25	50	50	NA	NA	NA
Norfloxacine	10	10	10	10	NA	NA	NA
Nystatin	NA	NA	NA	NA	100	100	100
Greseofulvin	NA	NA	NA	NA	500	100	100

Where: E.C., *Escherichia coli*; P.A., *Pseudomonas aeruginosa*; S.A., *Staphylococcus aureus*; S.P., *Staphylococcus pyogenes*; C.A., *Candida albicans*; A.N., *Aspergillus niger*; A.C., *Aspergillus clavatus*; MIC, minimum inhibitory concentration; MFCs, minimum fungicidal concentration; NS, not sensitive; NA, not applicable.

## Data Availability

Not applicable.

## Supplementary Materials

The following supporting information can be downloaded at: https://www.mdpi.com/article/10.3390/molecules28020501/s1, Table S1. The molecular interactions of designed derivatives with DHFR, Figure S1. Mass Spectrum of Synthesized Compound **27**, Figure S2. Mass Spectrum of Synthesized Compound **29**, Figure S3. Mass Spectrum of Synthesized Compound **30**, Figure S4. Mass Spectrum of Synthesized Compound **33**, Figure S5. Mass Spectrum of Synthesized Compound **37**, Figure S6. Mass Spectrum of Synthesized Compound **38**, Figure S7. Mass Spectrum of Synthesized Compound **41**.
