# Supplementary material for: Fighting Antibiotic Resistance: New Pyrimidine-Clubbed Benzimidazole Derivatives as Potential DHFR Inhibitors
Source: Molecules. 2023 Jan 4;28(2):501. doi: 10.3390/molecules28020501 (PMC9865878; doi:10.3390/molecules28020501)
Supplement: Supplementary file 1 [file molecules-28-00501-s001.zip › molecules-2113495-supplementary.pdf]

Article

Fighting antibiotic resistance: New pyrimidine-clubbed benzimidazole derivatives as potential DHFR inhibitors

M. Akiful Haque [1], Akash Marathakam [2], Ritesh Rana [3], Samar J Almehmadi [4], Vishal B. Tambe [5], Manoj Charde [6], Fahadul Islam [7], Falak A. Siddiqui [8], Giulia Culletta [9], Anna Maria Almerico [9], Marco Tutone [9]*, Sharuk L. Khan [8]*

[1] Department of Pharmaceutical Analysis, School of Pharmacy, Anurag University, Ghatkesar, Hyderabad, India-500088; akif963@gmail.com (M.A.H)
[2] National College of Pharmacy, Kozhikode, Kerala-673602, India; amarathakam@gmail.com (A.M)
[3] Department of Pharmaceutics, Himachal Institute of Pharmaceutical Education and Research (HIPER), Bela, Nadaun, Hamirpur, Himachal Pradesh, 177042, India; riteshpharma1719@gmail.com (R.R)
[4] Department of Chemistry, Faculty of Applied Science, Umm-Al-Qura University, Makkah, Saudi Arabia; sgmehmadi@uqu.edu.sa (S.J.A)
[5] Department of Pharmaceutical Chemistry, Pravara Rural College of Pharmacy, Loni, Maharashtra 413736, India; Email: vishaltambe55@gmail.com (V.B.T)
[6] Department of Pharmaceutical Chemistry, Government College of Pharmacy, Karad, Maharashtra 415124, India; Email: manojudps@rediffmail.com (M.C)
[7] Department of Pharmacy, Faculty of Allied Health Sciences, Daffodil International University, Dhaka 1207, Bangladesh; Email: fahadulislamdiu@gmail.com (F.I)
[8] Department of Pharmaceutical Chemistry, N.B.S. Institute of Pharmacy, Ausa 413520, Maharashtra, India; sharique.4u4@gmail.com (S.L.K); falakarjumand26@gmail.com (F.A.S)
[9] Dipartimento di Scienze e Tecnologie Biologiche Chimiche e Farmaceutiche, Università degli Studi di Palermo, 90123 Palermo, Italy; Email: culletta.giulia@gmail.com (G.C.); annamaria.almerico@unipa.it (A.M.A); marco.tutone@unipa.it (M.T.)

* Correspondence: sharique.4u4@gmail.com (S.L.K); marco.tutone@unipa.it (M.T.)

Table S1. The molecular interactions of designed derivatives with DHFR

Figure S1. Mass Spectrum of Synthesized Compound 27

Figure S2. Mass Spectrum of Synthesized Compound 29

Figure S3. Mass Spectrum of Synthesized Compound 30

Figure S4. Mass Spectrum of Synthesized Compound 33

Figure S5. Mass Spectrum of Synthesized Compound 37

Figure S6. Mass Spectrum of Synthesized Compound 38

Figure S7. Mass Spectrum of Synthesized Compound 41

Table S1. The molecular interactions of designed derivatives with DHFR

Active amino acid	Bond length	Bond type	Bond Category	Binding energy	Docking score
**Native ligand**					
**ASP27**	1.88237	Hydrogen bond	Conventional hydrogen bond	209.71	-8.5
**ASP27**	2.19462				
**ALA6**	3.00495				
**ILE5**	1.91594				
**ARG57**	1.96549				
**ARG57**	2.17225				
**ILE94**	3.19208		Carbon hydrogen bond		
**ILE50**	3.71343	Hydrophobic	Pi-Sigma		
**PHE31**	5.0747		Pi-Pi T-shaped		
**PHE31**	4.82737				
**ILE94**	4.98884		Alkyl		
**ILE5**	5.06209		Pi-Alkyl		
**ALA7**	4.05078				
25					
**GLY15**	3.55047	Hydrogen Bond	Carbon Hydrogen Bond	470.6	-7.2
**ALA7**	3.53102				
**GLY15**	3.56798				
**GLY95**	3.56301				
**GLY96**	3.62775				
**LEU28**	3.69758	Hydrophobic	Pi-Sigma		
**ILE50**	5.308		Alkyl		
**LEU28**	4.67657				
**MET20**	4.73921		Pi-Alkyl		
**LEU28**	5.28802				
**MET20**	5.1395				
**26**					
**ALA7**	3.49842	Hydrogen Bond	Carbon Hydrogen Bond	587.12	-8.5
**LEU28**	3.93756	Hydrophobic	Pi-Sigma		
**LEU28**	3.78875				
**PHE31**	4.56793		Pi-Pi T-shaped		
**ILE50**	5.31688		Alkyl		
**LEU28**	4.80815				
**MET20**	4.84878		Pi-Alkyl		
**LEU28**	5.18977				
**MET20**	5.32265				

**ILE5**	5.29874				
**ALA7**	4.42874				
		**27**			
**LEU28**	3.96052	Hydrophobic	Pi-Sigma	588.4	-8.6
**LYS32**	3.58775		Alkyl		
**LEU28**	4.33004		Pi-Alkyl		
**ALA7**	4.25134				
**PHE31**	4.87554				
		**28**			
**GLY15**	3.73566	Hydrogen Bond	Carbon Hydrogen Bond	588.98	-8.4
**ASP27**	3.17092	Halogen	Halogen (Fluorine)		
**ASP27**	3.60693				
**ASP27**	3.87889	Electrostatic	Pi-Anion		
**LEU28**	3.76687	Hydrophobic	Pi-Sigma		
**LEU28**	3.97034				
**LYS32**	3.61835		Alkyl		
**ALA7**	4.24717		Pi-Alkyl		
**PHE31**	4.82023				
		**29**			
**ALA7**	3.46744	Hydrogen Bond	Carbon Hydrogen Bond	589.31	-9.3
**LEU28**	3.93237	Hydrophobic	Pi-Sigma		
**LEU28**	3.75872				
**PHE31**	4.58907		Pi-Pi T-shaped		
**ILE50**	5.22086		Alkyl		
**LEU28**	4.7552				
**ILE5**	4.57366				
**ALA7**	4.25461				
**MET20**	4.94999		Pi-Alkyl		
**LEU28**	5.15219				
**MET20**	5.31113				
**ILE5**	5.26508				
**ALA7**	4.46628				
**TRP30**	5.07411				
		**30**			
**ALA7**	3.54199	Hydrogen Bond	Carbon Hydrogen Bond	589.91	-9.6
**LEU28**	3.97283	Hydrophobic	Pi-Sigma		
**LEU28**	3.8096				
**PHE31**	4.51892		Pi-Pi T-shaped		
**ILE50**	5.22229		Alkyl		

**LEU28**	4.75714				
**ILE5**	4.54982				
**ALA7**	4.2748				
**MET20**	4.92106		Pi-Alkyl		
**LEU28**	5.19863				
**MET20**	5.29169				
**ILE5**	5.24046				
**ALA7**	4.50596				
**TRP30**	5.21682				
		**31**			
**PRO21**	3.05404	Hydrogen Bond	Conventional Hydrogen Bond	520.64	-8.2
**TRP22**	2.07363				
**ASN23**	2.52579				
**GLU17**	2.67449				
**GLU17**	2.78723				
**GLY15**	3.65443		Carbon Hydrogen Bond		
**MET20**	5.37113	Hydrophobic	Alkyl		
**ILE5**	4.2758				
**ALA7**	4.27882				
**ALA7**	4.89267		Pi-Alkyl		
**PHE31**	4.98391				
		**32**			
**ASP27**	2.57625	Hydrogen Bond	Conventional Hydrogen Bond	588.87	-8.3
**ASP27**	3.95287	Electrostatic	Pi-Anion		
**LEU28**	3.98413	Hydrophobic	Pi-Sigma		
**LYS32**	3.6357		Alkyl		
**LEU28**	4.3027		Pi-Alkyl		
**ALA7**	4.30063				
**PHE31**	4.80426				
		**33**			
**MET20**	3.0518	Hydrogen Bond	Conventional Hydrogen Bond	826.79	-9
**ILE94**	2.68072				
**ASP27**	3.72887		Carbon Hydrogen Bond		
**MET20**	3.78307	Hydrophobic	Pi-Sigma		
**PHE31**	5.67997		Pi-Pi T-shaped		
**PHE31**	5.73244				
**ILE50**	4.96304		Alkyl		
**LYS32**	3.95492				
**LEU54**	4.99074				

**LEU28**	5.28126		Pi-Alkyl		
**ILE50**	4.93483				
**LEU28**	4.63308				
**ILE14**	5.34255				
**PHE31**	4.91521				
		**34**			
**ILE94**	2.86179	Hydrogen Bond	Conventional Hydrogen Bond	518.55	-8
**TRP22**	1.9705				
**TRP22**	2.35691				
**MET20**	4.3483	Other	Pi-Sulfur		
**PHE31**	4.86059	Hydrophobic	Pi-Pi T-shaped		
**ALA6:ALA7**	4.36561		Amide-Pi Stacked		
**MET20**	5.22922		Alkyl		
**ILE5**	4.79331				
**ALA7**	3.97847				
**ALA7**	5.28805		Pi-Alkyl		
**MET20**	5.45896				
**ILE5**	5.34592				
**ALA7**	4.54534				
		**35**			
**GLU17**	5.23367	Electrostatic	Attractive Charge	650.26	-8.2
**ILE94**	2.49803	Hydrogen Bond	Conventional Hydrogen Bond		
**ALA7**	3.05679				
**LEU28**	3.83897	Hydrophobic	Pi-Sigma		
**LEU28**	3.72044				
**PRO25**	4.0166		Alkyl		
**ILE5**	4.55375				
**ALA7**	4.09288				
**PHE31**	4.97521		Pi-Alkyl		
		**36**			
**ASP27**	3.64005	Hydrogen Bond	Carbon Hydrogen Bond	544.97	-7.9
**ALA7**	3.48216				
**GLY15**	3.50798				
**GLU17**	4.88488	Electrostatic	Pi-Anion		
**LEU28**	3.7657	Hydrophobic	Pi-Sigma		
**MET20**	5.71901	Other	Pi-Sulfur		
**MET20**	5.75583				
**PHE31**	4.72955	Hydrophobic	Pi-Pi T-shaped		
**LEU28**	5.34773		Alkyl		
**ILE50**	5.22702				
**LEU28**	5.4916		Pi-Alkyl		

**ILE5**	5.16431				
**ALA7**	4.16746				
			**37**		
**ALA7**	2.56058	Hydrogen Bond	Conventional Hydrogen Bond	605.87	-8.7
**ASP27**	3.62684		Carbon Hydrogen Bond		
**TYR100**	5.20995	Hydrophobic	Pi-Pi T-shaped		
**ILE50**	5.0427		Alkyl		
**MET20**	4.83558		Pi-Alkyl		
**MET20**	4.53183				
**LEU28**	5.48553				
**ILE14**	4.73176				
			**38**		
**GLU17**	4.78972	Electrostatic	Attractive Charge	705.74	-9
**ILE94**	2.50758	Hydrogen Bond	Conventional Hydrogen Bond		
**LEU28**	3.94835	Hydrophobic	Pi-Sigma		
**MET20**	5.89342	Other	Pi-Sulfur		
**PHE31**	5.00102	Hydrophobic	Pi-Pi T-shaped		
**ALA6:ALA7**	4.55365		Amide-Pi Stacked		
**ILE5**	4.83846		Alkyl		
**ALA7**	3.94521				
**MET20**	5.46796		Pi-Alkyl		
**ALA7**	4.74961				
**LEU28**	5.15282				
**MET20**	4.85642				
**PHE31**	5.29782				
			**39**		
**ARG57**	2.58638	Hydrogen Bond	Conventional Hydrogen Bond	937.76	-8.3
**ARG57**	2.18282				
**PHE31**	4.7992	Hydrophobic	Pi-Pi T-shaped		
**MET20**	5.18068		Alkyl		
**ILE5**	4.84697				
**ALA7**	4.48924				
**MET20**	5.37667		Pi-Alkyl		
**ILE5**	5.45489				
**ALA7**	4.556				
**LEU28**	5.02873				
**LEU54**	5.45716				
**TRP30**	5.46865				
**PHE31**	5.01022				

			**40**		
**GLU17**	5.19129	Electrostatic	Attractive Charge	622.83	-7.1
**SER49**	3.54558	Hydrogen Bond	Carbon Hydrogen Bond		
	4.54671	Hydrophobic	Pi-Pi Stacked		
**PHE31**	5.46322				
	3.75304				
**PHE31**	4.10963				
**LEU28**	5.15441		Pi-Alkyl		
**ILE50**	5.32509				
**ILE50**	5.13593				
**MET20**	5.13796				
			**41**		
**THR113**	2.73475	Hydrogen Bond;Halogen	Conventional Hydrogen Bond;Halogen (Fluorine)	596.44	-9
**TRP30**	3.57593	Hydrogen Bond	Carbon Hydrogen Bond		
**ILE5**	3.54683	Halogen	Halogen (Fluorine)		
**ALA6**	3.42515				
**ALA6**	3.17175				
**ASP27**	3.59013				
**ASP27**	2.97815				
**ASP27**	3.08315				
**ASP27**	3.22541				
**LEU28:**	3.98408	Hydrophobic	Pi-Sigma		
**LYS32**	3.49227		Alkyl		
**ILE5**	4.58651				
**ALA7**	4.22775				
**LEU28**	4.3502		Pi-Alkyl		
**ALA7**	4.17323				
**PHE31**	4.91107				
**PHE31**	5.25311				

Figure S1. Mass Spectrum of Synthesized Compound 27

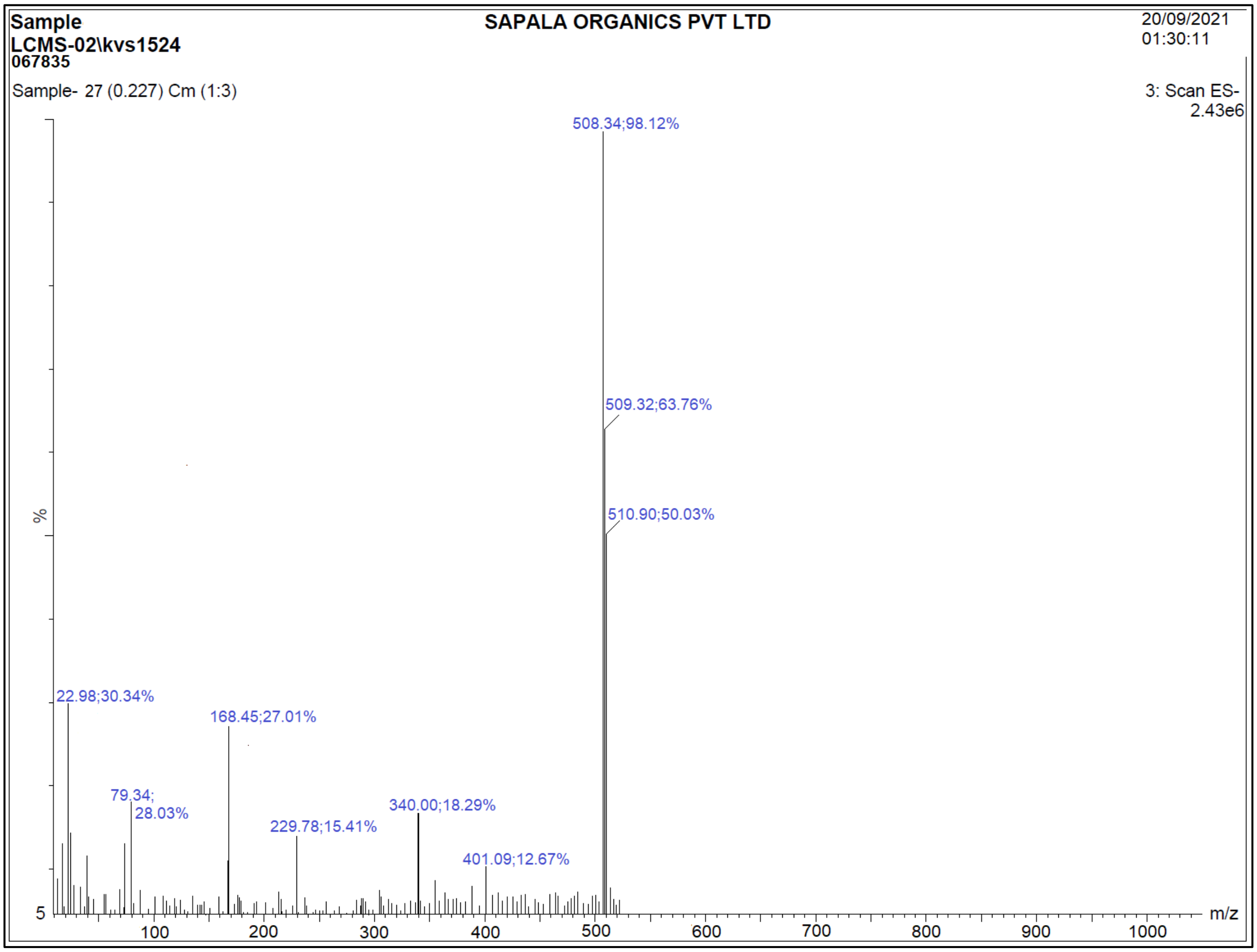

Methyl 2-((6-chloro-1H-benzo[d]imidazol-2-yl)methylthio)-4-(4-bromophenyl)-1,2,3,4-tetrahydro-6-methylpyrimidine-5-carboxylate **(27)**

Figure S2. Mass Spectrum of Synthesized Compound 29

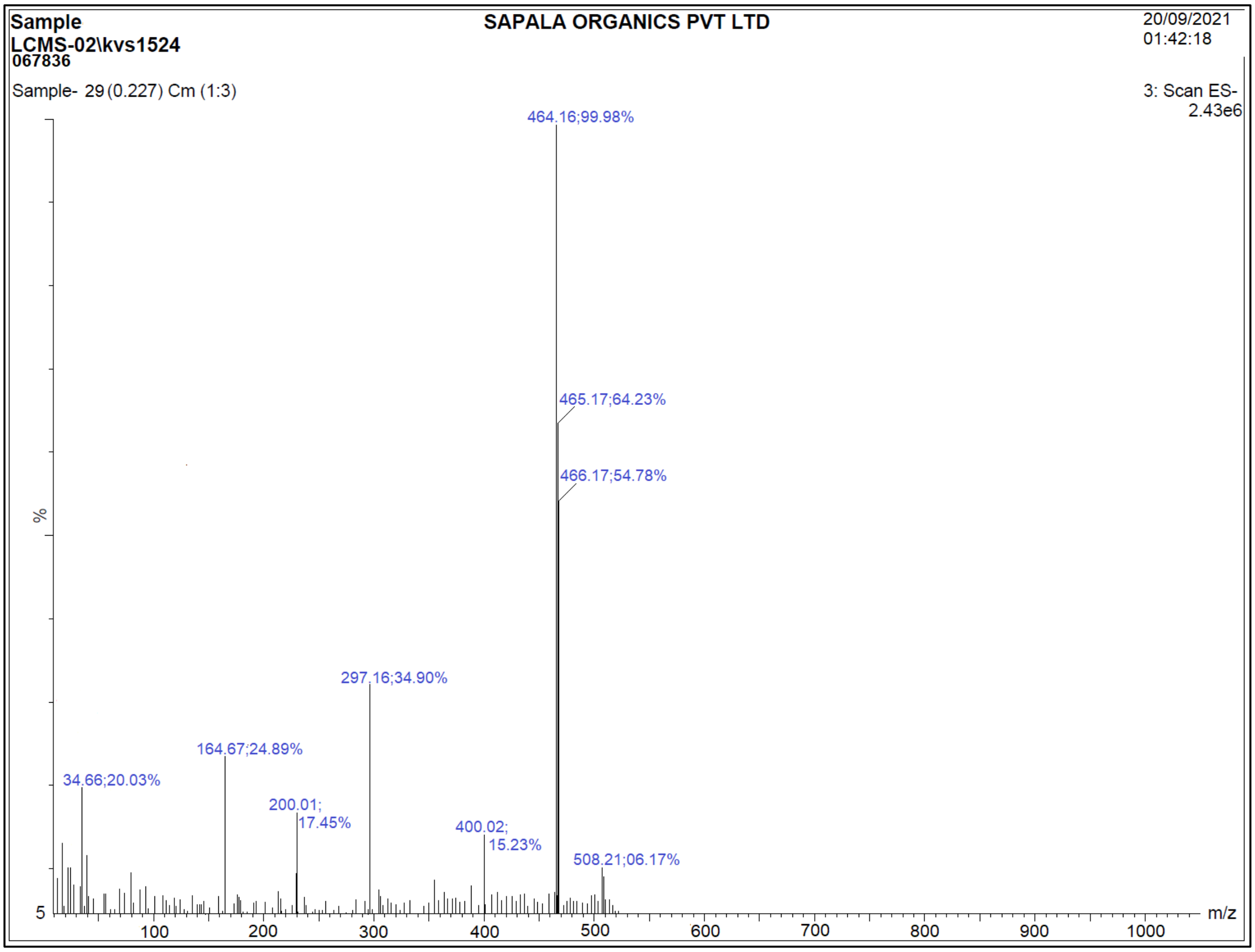

Methyl 2-((6-chloro-1H-benzo[d]imidazol-2-yl) methylthio)-4-(4-chlorophenyl)-1, 2, 3, 4-tetrahydro-6-methylpyrimidine-5-carboxylate (**29**)

Figure S3. Mass Spectrum of Synthesized Compound 30

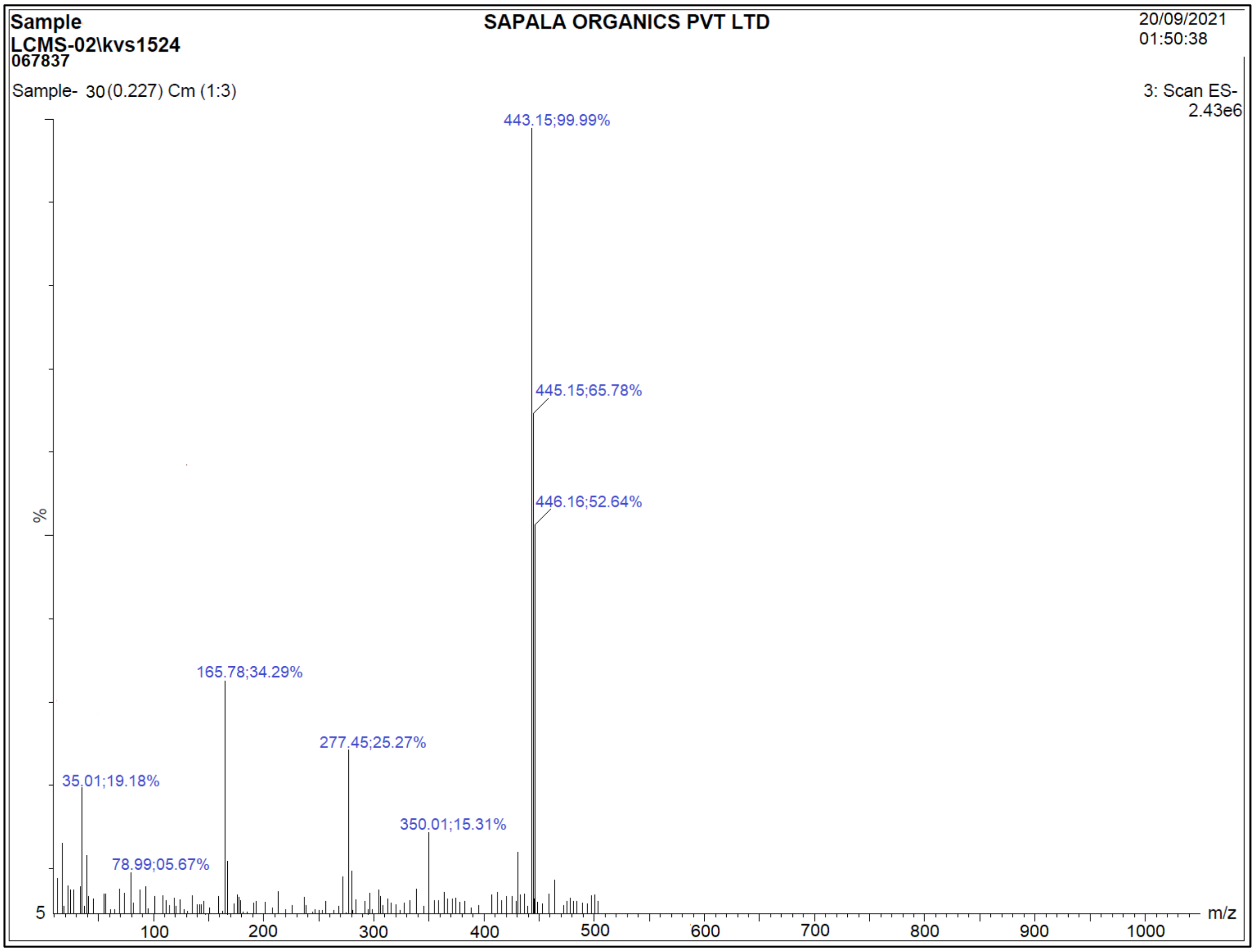

Methyl 2-((6-chloro-1H-benzo[d]imidazol-2-yl)methylthio)-1,2,3,4-tetrahydro-6-methyl-4-p-tolylpyrimidine-5-carboxylate **(30)**

Figure S4. Mass Spectrum of Synthesized Compound 33

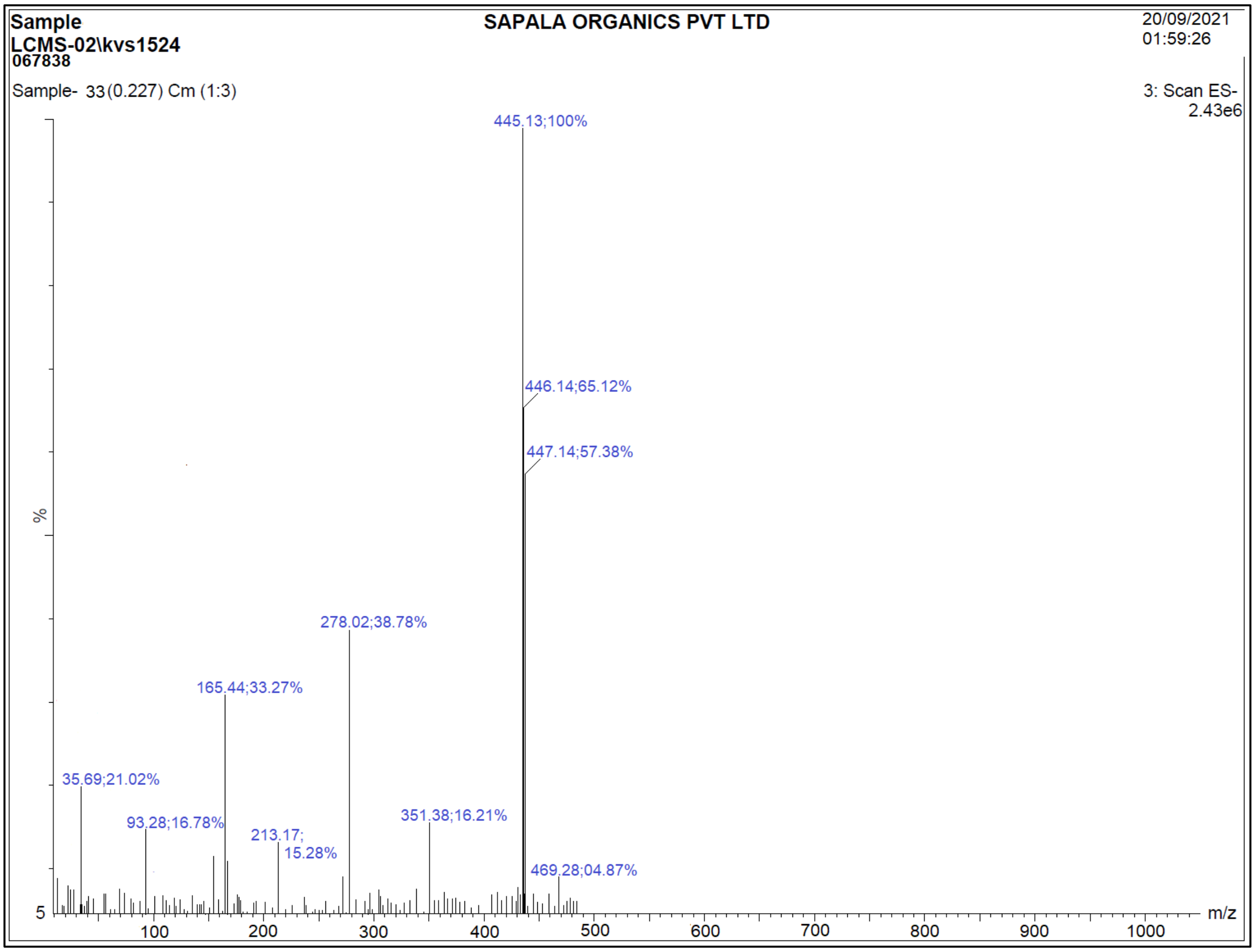

Methyl 2-((6-chloro-1H-benzo[d]imidazol-2-yl)methylthio)-1,2,3,4-tetrahydro-4-(3-hydroxyphenyl)-6-methylpyrimidine-5-carboxylate **(33)**

Figure S5. Mass Spectrum of Synthesized Compound 37

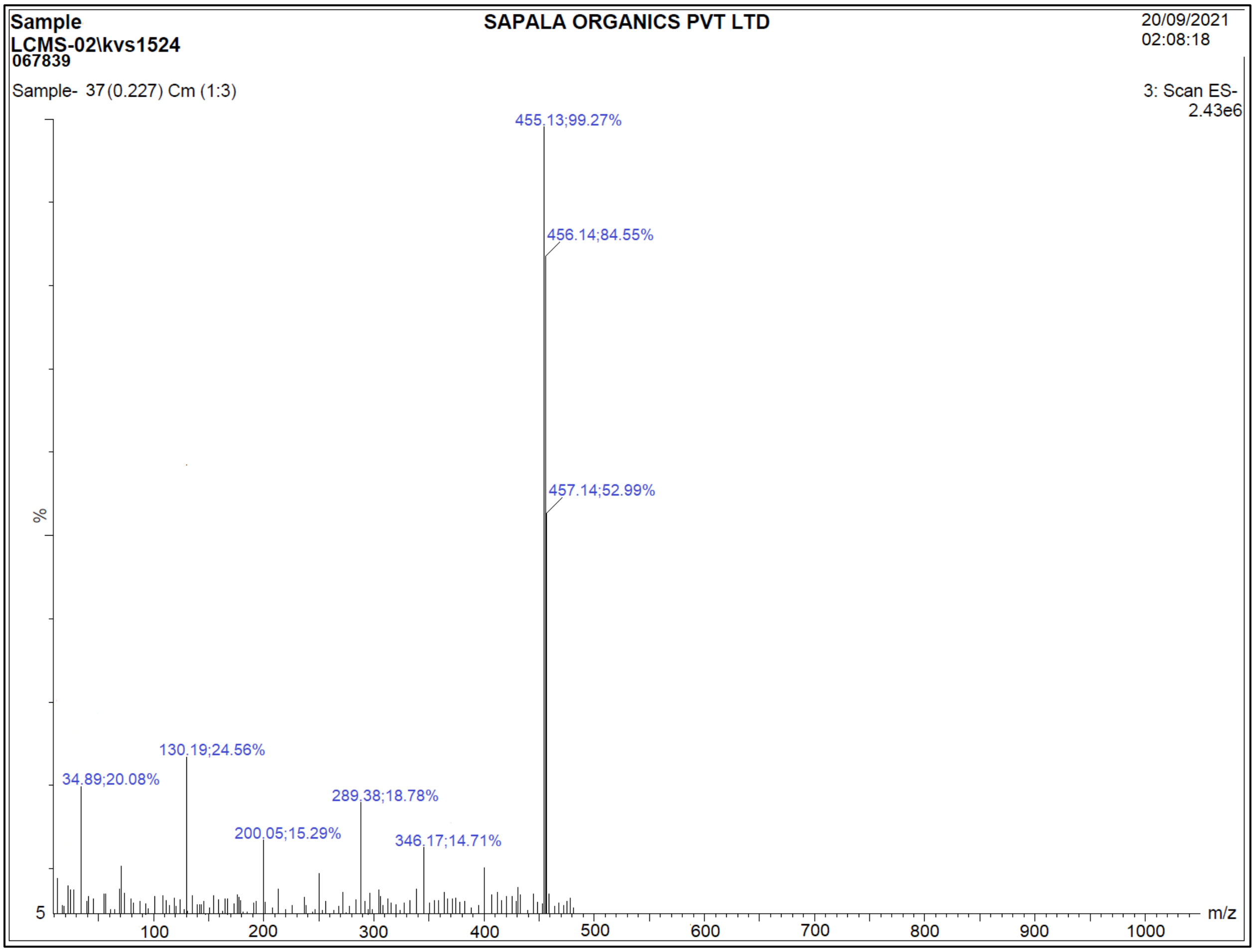

Methyl 2-((6-chloro-1H-benzo[d]imidazol-2-yl)methylthio)-1,2,3,4-tetrahydro-6-methyl-4-styrylpyrimidine-5-carboxylate **(37)**

Figure S6. Mass Spectrum of Synthesized Compound 38

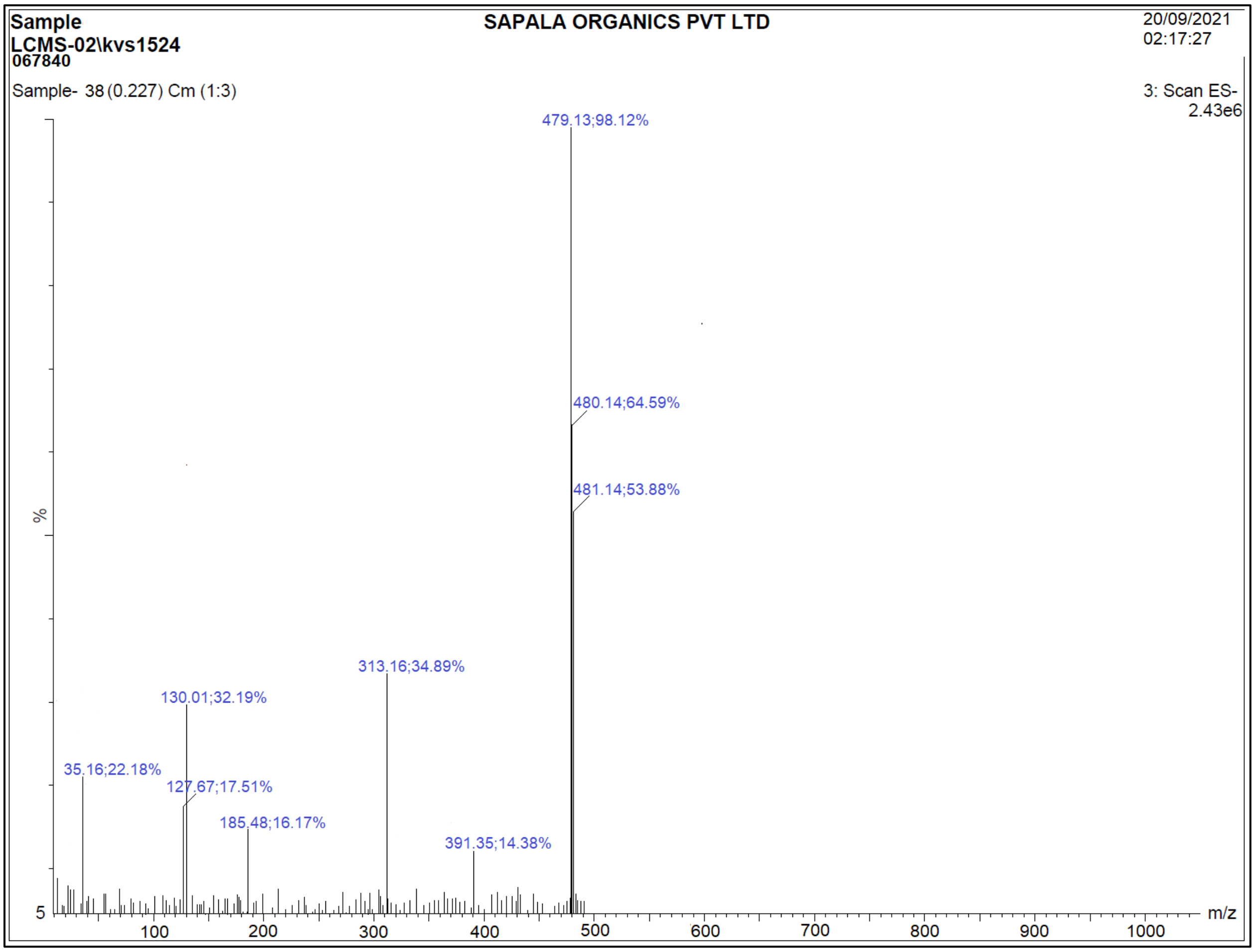

Methyl 2-((6-chloro-1H-benzo[d]imidazol-2-yl)methylthio)-1,2,3,4-tetrahydro-6-methyl-4-(naphthalen-1-yl)pyrimidine-5-carboxylate **(38)**

Figure S7. Mass Spectrum of Synthesized Compound 41

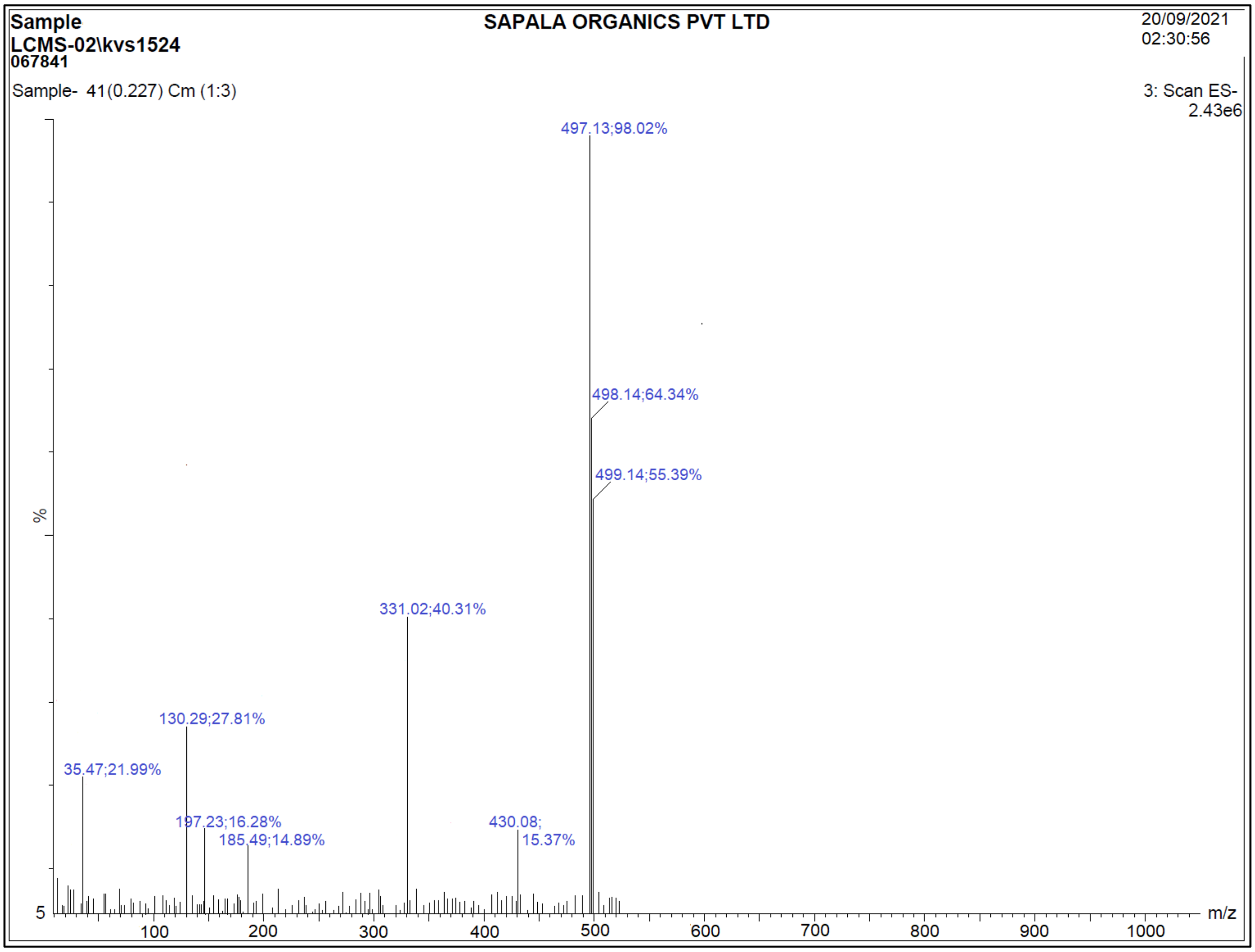

Methyl 2-((6-chloro-1H-benzo[d]imidazol-2-yl)methylthio)-4-(4-(trifluoromethyl)phenyl)-1,2,3,4-tetrahydro-6-methylpyrimidine-5-carboxylate **(41)**
